# Core microbiota drive multi-functionality of the soil microbiome in the *Cinnamomum camphora* coppice planting

**DOI:** 10.1186/s12866-023-03170-8

**Published:** 2024-01-10

**Authors:** Luyuan Sun, Guilong Li, Jiao Zhao, Ting Zhang, Jia Liu, Jie Zhang

**Affiliations:** 1 Jiangxi Provincial Engineering Research Center for Seed- breeding and Utilization of Camphor Trees, Nanchang Institute of Technology, Nanchang, 330099 China; 2 Soil and Fertilizer & Resources and Environment Institute, Jiangxi Academy of Agricultural Sciences, Nanchang, 330200 China; 3 Jiangxi Academy of Forestry, Nanchang, 330032 China

**Keywords:** Core microbiota, *Cinnamomum camphora*, Ecosystem multi-functionality, Tree planting, Coppice soils

## Abstract

**Background:**

*Cinnamomum camphora* (L.) Presl (*C. camphora*) is an evergreen broad-leaved tree cultivated in subtropical China. The use of *C. camphora* as clonal cuttings for coppice management has become popular recently. However, little is known about the relationship between soil core microbiota and ecosystem multi-functionality under tree planting. Particularly, the effects of soil core microbiota on maintaining ecosystem multi-functionality under *C. camphora* coppice planting remained unclear.

**Materials and methods:**

In this study, we collected soil samples from three points (i.e., the abandoned land, the root zone, and the transition zone) in the *C. camphora* coppice planting to investigate whether core microbiota influences ecosystem multi-functions.

**Results:**

The result showed a significant difference in soil core microbiota community between the abandoned land (AL), root zone (RZ), and transition zone (TZ), and soil ecosystem multi-functionality of core microbiota in RZ had increased significantly (by 230.8%) compared to the AL. Soil core microbiota played a more significant influence on ecosystem multi-functionality than the non-core microbiota. Moreover, the co-occurrence network demonstrated that the soil ecosystem network consisted of five major ecological clusters. Soil core microbiota within cluster 1 were significantly higher than in cluster 4, and there is also a higher *Copiotrophs/Oligotrophs* ratio in cluster 1. Our results corroborated that soil core microbiota is crucial for maintaining ecosystem multi-functionality. Especially, the core taxa within the clusters of networks under tree planting, with the same ecological preferences, had a significant contribution to ecosystem multi-functionality.

**Conclusion:**

Overall, our results provide further insight into the linkage between core taxa and ecosystem multi-functionality. This enables us to predict how ecosystem functions respond to the environmental changes in areas under the *C. camphora* coppice planting. Thus, conserving the soil microbiota, especially the core taxa, is essential to maintaining the multiple ecosystem functions under the *C. camphora* coppice planting.

**Supplementary Information:**

The online version contains supplementary material available at 10.1186/s12866-023-03170-8.

## Introduction

Soils are living ecosystems forming the foundation of terrestrial ecosystems, with multiple ecological functions, including maintaining sustainable agricultural production and environmental well-being [1, 2]. However, global climate change and anthropogenic activities are exerting significant pressure on the natural environment surrounding the agricultural landscape, resulting in soil quality degradation and species loss [3]. As a part of broader restoration efforts and to improve soil health and functions, tree planting has been applied globally [4]. It is suggested that planting more trees can create more diverse niches for underground microbes to thrive [5, 6]. Indeed, an increasing number of studies support the argument that tree planting can significantly influence the diversity and composition of soil microbiota directly by modifying soil properties and resource utilization patterns [7, 8]. However, some studies suggest that tree planting may have adverse effects. Particularly, some tree species can alter soil structure and water distribution in the soil column, negatively influencing the stability and resilience of restored ecosystems [9, 10]. Therefore, understanding the variation in soil microbiota in agroecosystems under tree planting is of great significance for improving soil health and functions and achieving sustainable agricultural production [11].

Soil microbiomes are highly complex and diverse, and they are involved in multiple important ecological and physiological functions (hereafter, multi-functionality [12, 13]), including soil organic matter decomposition and cycling, influence the availability of mineral nutrients, nitrogen fixation, and primary productivity [14–16]. Recently, links between soil biodiversity and ecosystem multi-functionality have received increasing attention, and studies have reported positive relationships between higher levels of microbial abundance and ecosystem multi-functionality [17, 18]. Nevertheless, soil ecosystem multi-functionality among microorganisms may vary with microbial functional groups, with certain groups having greater effects than others [19, 20].

Microbiota are present in large numbers across all ecosystems and are regarded as “core taxa” due to their significant influence on soil community functioning [21, 22]. However, the role of the core microbiota in maintaining the ecosystem multi-functionality remains unexplored [23]. Recent studies also have shown that soil organisms tend to coexist and form distinct ecological clusters of specific taxa [24, 25]. These taxa within the clusters are anticipated to interact and perform multiple functions, such as maintaining soil quality and enhancing agriculture production [26, 27]. As a result, ecological clusters could potentially form based on the core soil taxa that contribute to multiple ecosystem functions. Unfortunately, to our knowledge, no previous studies have examined the correlation between the multiple ecosystem functions and the ecological clusters of soil core groups under tree plantation.

*Cinnamomum camphora* (L.) Presl is a broad-leaved evergreen tree from the Lauraceae family with a wide distribution in subtropical China [7]. The tree contains many secondary metabolites, and the whole plant has traditionally been felled to harvest the roots, stems, and leaves and extract essential oil [28–30]. In recent years, the *C. camphora* tree has been utilized for coppice management, particularly in southern China [31, 32]. Then, the aerial parts of the *C. camphora* coppice were cut down at a distance of 20 cm above the ground from July to September annually. Unlike traditional tree planting, the remaining part of the tree stump regenerates year over year, leading to the circular production of *C. camphora* coppice, parallel to managing perennial crops. Despite the popularity of coppice management, few studies have examined the impact of coppice management practice on soil core microbiota and ecosystem multi-functionality.

To enrich our cognition on how soil core microbiota and ecosystem multifunctionality respond to the *C. camphora* coppice plantation, we collected the top 15 cm soils from the five-year *C. camphora* coppice plantation land to make further analysis. Soil samples were collected from three different points, the abandoned land (AL), the root zone (RZ), and the transition zone (TZ). Particularly, we asked the following questions: (1) how *C. camphora* coppice planting affects soil core microbiota, (2) how *C. camphora* coppice planting impacts soil ecosystem multi-functionality, and (3) what is the relationship between soil microbiota and ecosystem multi-functionality under *C. camphora* coppice planting? This study emphasized the significance of soil core microbiota and ecosystem multi-functionality in agroecosystems, which can ultimately offer a theoretical basis for scientifically guiding the production and management of *C. camphora* coppice plantations in subtropical China.

## Materials and methods

### Study area

The field experiment was conducted on *C. camphora* coppice plantation land in Guixi City, Jiangxi Province, southern China (28°17′46″ N, 117°13′28″E). The research site has a subtropical monsoon climate with a mean annual temperature of 18.8℃. The experimental site receives a mean annual precipitation of 1980.8 mm and 1611.5 h of sunlight, respectively. The experimental soils belong to Ultisols, which was abandoned land before. At the beginning of the experiment, the topsoil (0–15 cm) had a soil pH of 4.83, soil organic carbon (SOC) of 12.95 g·kg^− 1^, soil total nitrogen (TN) of 0.96 g·kg^− 1^, soil available nitrogen (AN) of 102.93 g·kg^− 1^ and soil available phosphorus (AP) of 5.21 g·kg^− 1^.

### Experiment design and soil sampling

**Fig. 1:**
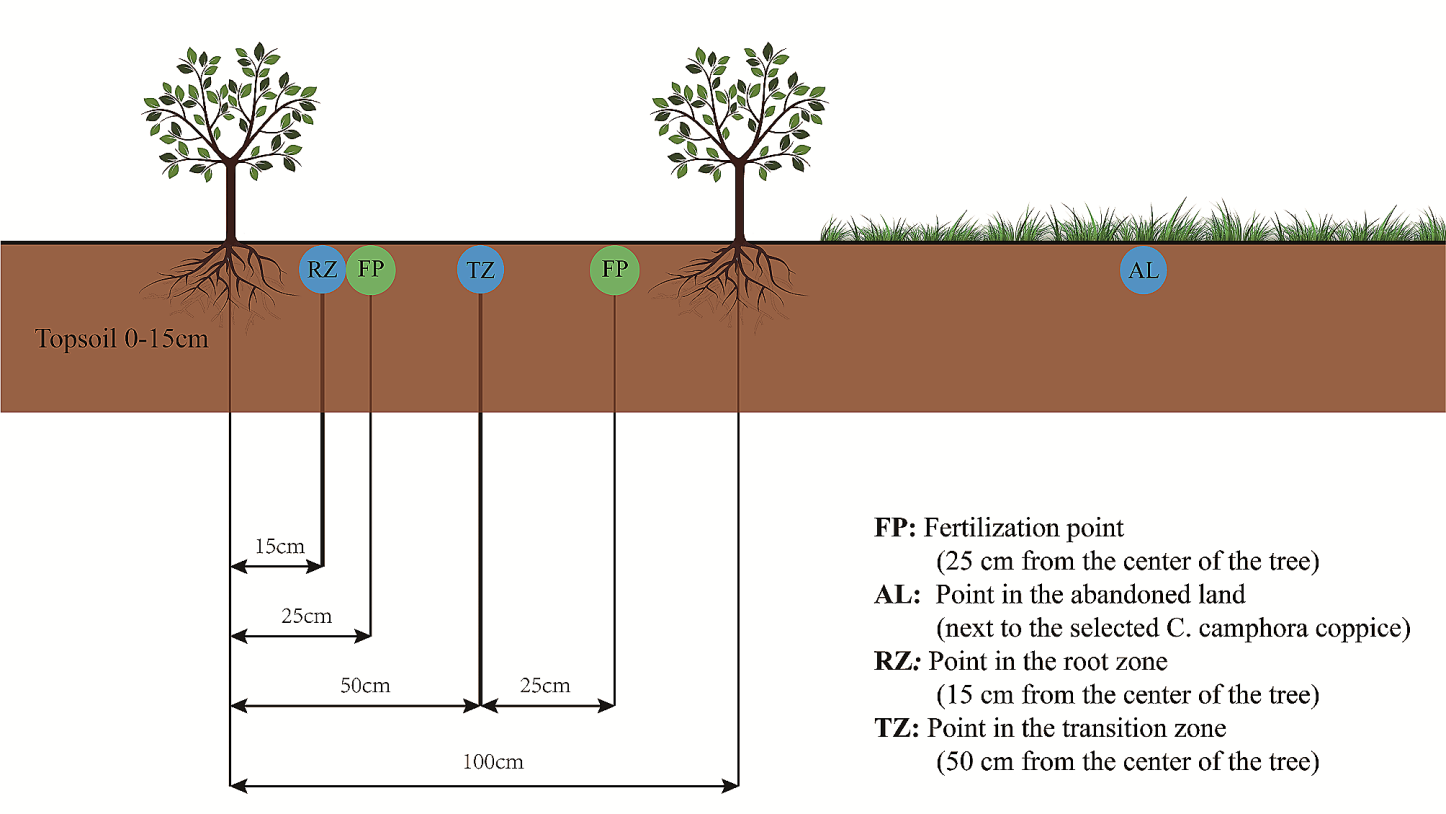
Experiment layout with sampling details

In this study, we selected a 5000 m^2^*C. camphora* coppice plantation land established in 2015. The initial plantation land is dominated by species *Firmiana simplex*, *Broussonetia papyrifera*, *Phytolacca acinose*, *Humulus scanden*s, *Eleusine indica*, *Duchesnea indica*, and *Erigeron canadensis*. Before *C. camphora* coppice planting, vegetation growing on this land should be cleared first, and then manual weed control will be carried out regularly every year. And we don’t use any weeding measures on the abandoned land (AL). Every year since 2016, in early September, we cut down the *C. camphora* tree from 20 cm above the ground to extract the essential oil. The row and inter-plant spacings of the *C. camphora* tree was 1 m × 1 m, with a planting density of 10,000 trees·hm^− 2^. The coppice plantation was fertilized, with an application rate of 150 g tree^− 1^ year^− 1^ for N_2_O_5_, P_2_O_5_, and K_2_O. The fertilizers were applied in two phases, with 50% applied in early March and the remaining 50% after the *C. camphora* tree was felled in late September. The fertilization point (FP) was a 15 cm deep circle at a distance of 25 cm from the center of the tree (Fig. 1).

In this study, we selected three distinct locations as our sampling points, each with four replicates: (1) a point in the abandoned land (AL); (2) a point in the root zone (RZ); and (3) a point in the transition zone (TZ). From each point, Soil samples were randomly collected using the S-shaped sampling method after the *C. camphora* tree felling on September 12th, 2021. Soil samples from the top 15 cm were taken using a 2 cm diameter soil core sampler. Multiple soil samples (n = 20) were taken from a 50 m^2^ area and composited. In the Lab, the composited soil samples were passed through the 2 mm sieve separately to remove the impurities. The sieved soils for each sample were divided into three parts: one was air-dried to measure soil fertility, the second was preserved at 4℃ to measure enzyme activity, and the remaining was kept at -80℃ for DNA extraction.

### Soil properties and enzyme activity

After the soil was suspended with water at a ratio of 1:2.5 (weight:volume), the pH was measured with a pH meter (FE28-Standard, METTLER-TOLEDO, Switzerland). Soil organic carbon (SOC) was determined by dichromate oxidation and titration with ferrous sulfate. Soil total nitrogen (TN) was determined using the Kjeldahl digestion distillation method. Soil available nitrogen (AN) and phosphorus (AP) were determined using alkali hydrolyzation and the Bray method, respectively [33]. Soil enzyme activities of invertase (INV), urease (UE), acid phosphatase (ACP), catalase (CAT), polyphenol oxidase (PPO), and peroxidase (POD) were determined using kits from Beijing Solarbio Technology Co. Ltd according to the manufacturer’s instructions [34, 35].

### Soil microbial DNA extraction and Illumina MiSeq sequencing

DNA was extracted from 0.5 g of fresh soil using the FastDNA® SPIN Kit for Soil (MP Bio-medicals, Santa Ana, CA) following the manufacturer’s instructions. The purification of DNA was tested by Power Clean DNA Clean-Up Kit (MOBIO Laboratories, Carlsbad, CA, USA), and the PCR inhibitors were removed. The eluted DNA was examined by 1% (m/v) agarose gel electrophoresis and quantified using the NanoDrop® 2000 spectrophotometer (Thermo Fisher Scientific, USA). Aliquots of the DNA were stored in a − 20 °C freezer for subsequent analyses. The V4-V5 highly variable regions of the bacterial 16 S rRNA genes were amplified with barcoded universal primers 515 F/907R [36]. Moreover, the ITS regions of the fungal rRNA genes were amplified using primer sets ITS1/ITS2 [37].

### Bioinformatics analysis for raw sequences

Raw Illumina reads were analyzed using the QIIME v.1.91 pipeline [38]. The low-quality (quality score < 25) or short (length < 200 bp) sequences were removed before downstream analysis [39]. From the remaining high-quality sequences, Operational Taxonomic Units (OTUs) were identified from the high-quality sequences at a 3% dissimilarity level using the UNOISE algorithm [40]. The RDP classifier with the SILVA 132 database [41] was utilized to predict the taxonomic identity of the final stock with confidence estimates of 80% confidence estimate. The representative sequences of ITS were annotated for species using the blast method with the UNITE database [42]. A total of 4427 bacteria OTUs and 1690 fungal OTUs were then selected for subsequent downstream analysis.

### Statistical analyses

Statistical tests were performed by the Wilcoxon test and the Kruskal-Wallis test. Wilcoxon tests were used for within-group comparisons, and Kruskal Wallis tests were used for between-group comparisons. The core microbiota present in bacterial and fungal communities were identified using the following criteria: (1) the highly abundant OTUs with relative abundance in the top 10% were selected from all the soil samples, and (2) the ubiquitous OTUs were presented in 95% of the soil samples were retained. Thus, the selected OTUs under the two criteria were abundant and ubiquitous across the 12 soil samples in this study [43]. Principal Coordinates Analysis (PCoA) was used in the R packages phyloseq (v.1.32.0) to explore the differences in soil core microbiota community between the sampling points based on Bray–Curtis distances [44]. Significant differences in the overall community composition between the sampling points were examined statistically using analysis of similarity (ANOSIM). Analysis of differential OTUs was detected by the R packages edgeR (v.4.1.2) to identify the enriched OTUs in different sampling points [45].

Soil ecosystem multifunctionality was accessed by measuring: (1) soil nutrients cycling: AN and AP; (2) soil fertility: SOC and TN; and (3) soil activity: UE, CAT, ACP, CAT, PPO, and POD (Table S2). First, the normality of relevant soil data was checked using the Shapiro-Wilk test, and whenever necessary, variables were logarithm or square root transformed to meet normality and homogeneity assumptions [46]. Then, the average index approach was used to calculate the soil ecosystem multifunctionality [17, 47]. In order to ensure consistency in the scaling of the data, each variable was standardized with a range of zero to one. The average multifunctional index was then calculated by taking the mean value of indexes in each sampling point [48, 49]. A random forest model was used to predict the main drivers of soil multifunctionality with the R packages rfPermute (version 2.5) [50]. The significance of variables was determined through the percentage of mean squared error (MSE%): higher MSE% represents more significant variables [51].

The Weighted Gene Co-Expression Network Analysis (WGCNA) was used in the R packages WGCNA (v.1.70-3) to determine soil hub taxa [52]. The soft threshold power was determined using the scale-free topological criterion with an R^2^ of 0.9. The adjacency matrix was constructed according to the most appropriate soft threshold power of 10, which was then transformed into a topological overlap matrix (TOM). Then, TOM-based dissimilarity and dynamic branch cutting were used to identify different ecological clusters. The OTUs with high module membership values (greater than 0.9) were called hub taxa. The relative abundance of OTUs included in the clusters was normalized and then averaged to obtain the relative abundance of the cluster [27].

## Results

### The composition and structure of soil core microbiota in the *C. camphora* coppice planting

**Fig. 2:**
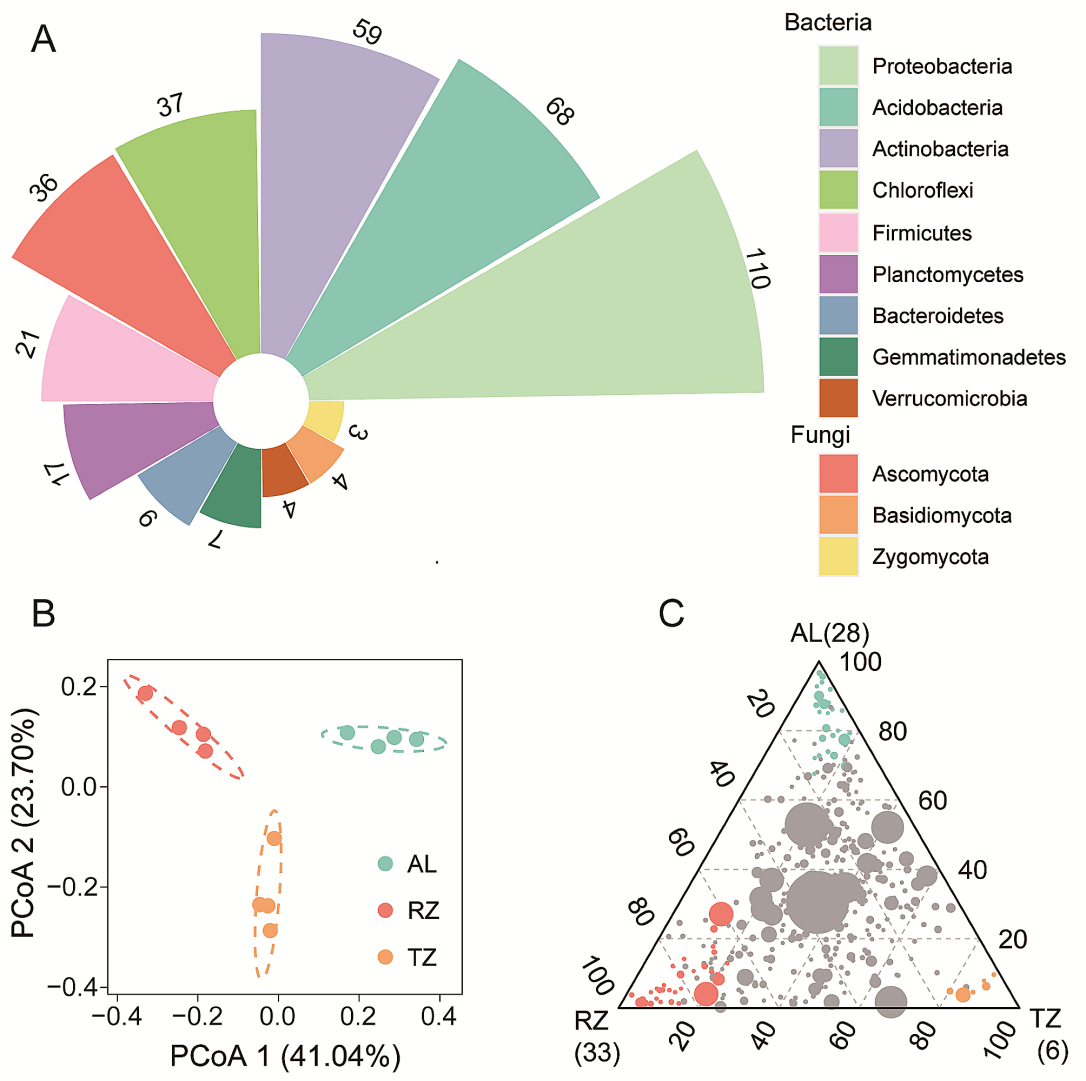
(**A**) The number of soil microbial taxa in each phylum under *C. camphora* planting were illustrated in the pie chart. (**B**) PCoA plot depicting the core microbiota in the *C. camphora* coppice planting based on Bray–Curtis distances. (**C**) Ternary plot of all OTUs detected in the dataset with a relative abundance > 5% in at least one soil sample in the *C. camphora* coppice planting, where each point corresponds to one out and the size of a point represents the average relative abundance, and the position depicted the contribution of the indicated compartments to the total relative abundance. Colored circles represent OTUs enriched in one compartment compared with the others; green in AL, orange in RZ, and red in TZ, whereas gray circles represent OTUs not significantly enriched in a specific compartment. Note: AL, abandoned land; RZ, root zone; and TZ, transition zone

Under the AL, RZ, and TZ, there were 651,537 and 748,374 soil bacterial and fungal sequences. After filtering and standardization, 4404 and 1409 OTUs in soil bacteria and fungi were clustered, respectively. The soil core microbiota were confirmed by selecting highly abundant (with relative abundance in the top 10%) and ubiquitous OTUs (presenting in 95% of all soil samples). The analysis of soil core taxa identified a total of 375 OTUs, consisting of 332 soil bacterial taxa and 43 soil fungal taxa. (Table S1). The soil core taxa were dominated by bacteria taxa, and the phylum Proteobacteria had the highest number (n = 110), followed by Acidobacteria (n = 68), Actinobacteria (n = 59), and Chloroflexi (n = 37). The fungal phylum mainly consisted of Ascomycota (n = 36), Basidiomycota (n = 4), and Zygomycota (n = 3). (Fig. 2A).

**Fig. 3:**
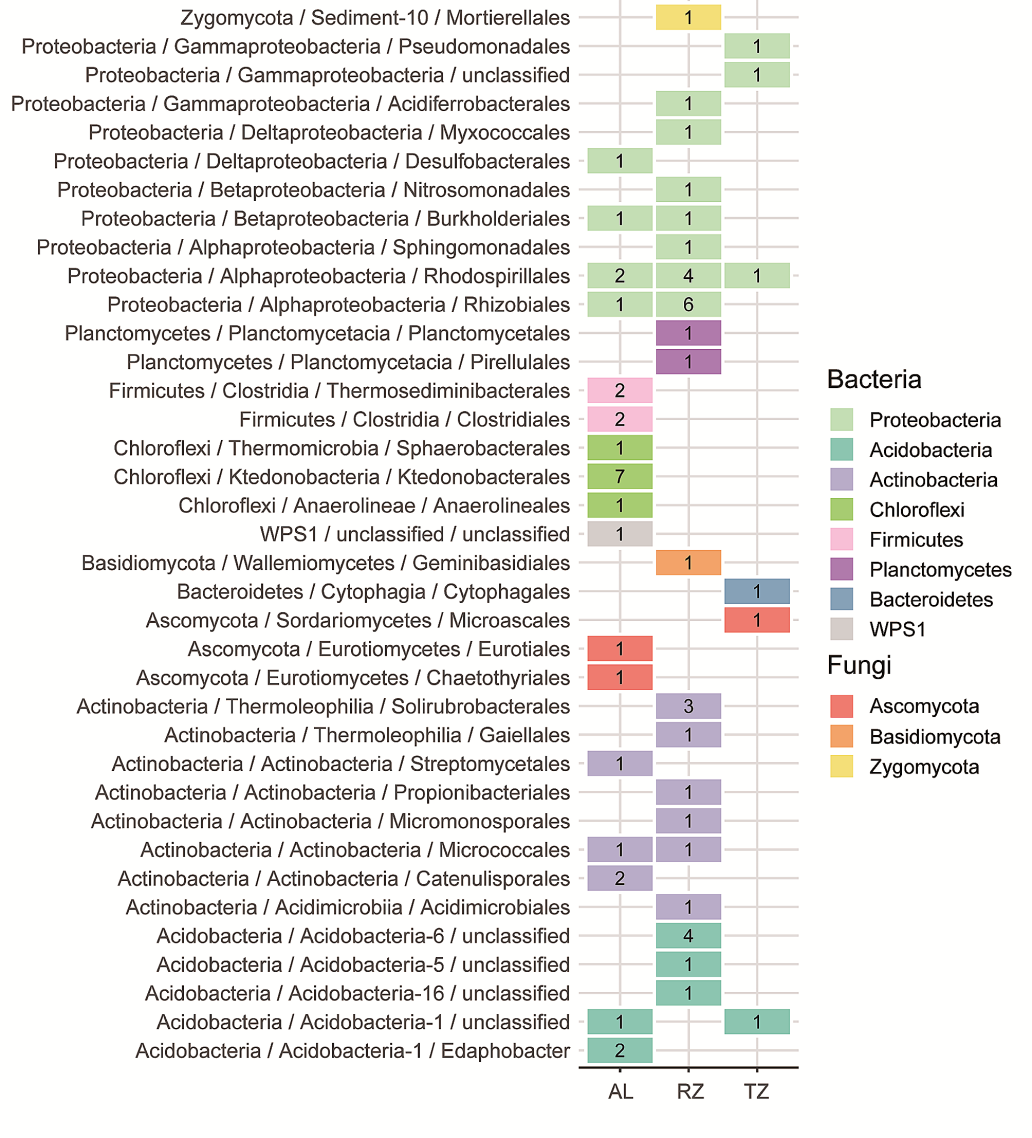
Heatmap of the number of soil core microbiota at phylum level in the *C. camphora* coppice planting. Note: AL, abandoned land; RZ, root zone; and TZ, transition zone

The PCoA analysis revealed that the soil core microbiota community was distinctly separated in ordination space according to the sampling points (Fig. 2B). The microbiota community in the AL was clustered separately from the RZ and TZ and along the first coordinate axis. Overall, PCoA explained 64.74% of the total variance of the soil core microbiota community composition in the *C. camphora* coppice planting, of which the first axis (PCoA 1) explained 41.04% of the variance, and the second constraint axis (PCoA 2) explained 23.70% of the variance (Fig. 2B). ANOSIM confirmed a significant difference in soil core microbiota community between the sampling points (*p* < 0.05, Table S2). The compositional difference was much more pronounced between AL with RZ (*R*_*ANOSIM*_ = 0.9272, *p* = 0.027), and AL and TZ (*R*_*ANOSIM*_ = 0.9271, *p* = 0.030) than between RZ and TZ (*R*_*ANOSIM*_ = 0.8438, *p* = 0.026). The ternary plot was used to illustrate OTUs that are specifically enriched between different sampling points, and results showed 28 OTUs enriched in AL, 33 OTUs enriched in RZ, and 6 OTUs enriched in TZ (Fig. 2C). Among the enriched OTUs in AL, Chloroflexi was the most dominant phylum, with 9 OTUs, accounting for 32.14% of the total OTUs. Proteobacteria was the most dominant phylum, with 15 OTUs in RZ, representing 45.45% of the total OTUs (Fig. 3).

### The effects of soil core microbiota on soil ecosystem multifunctionality in the *C. camphora* coppice planting

**Fig. 4:**
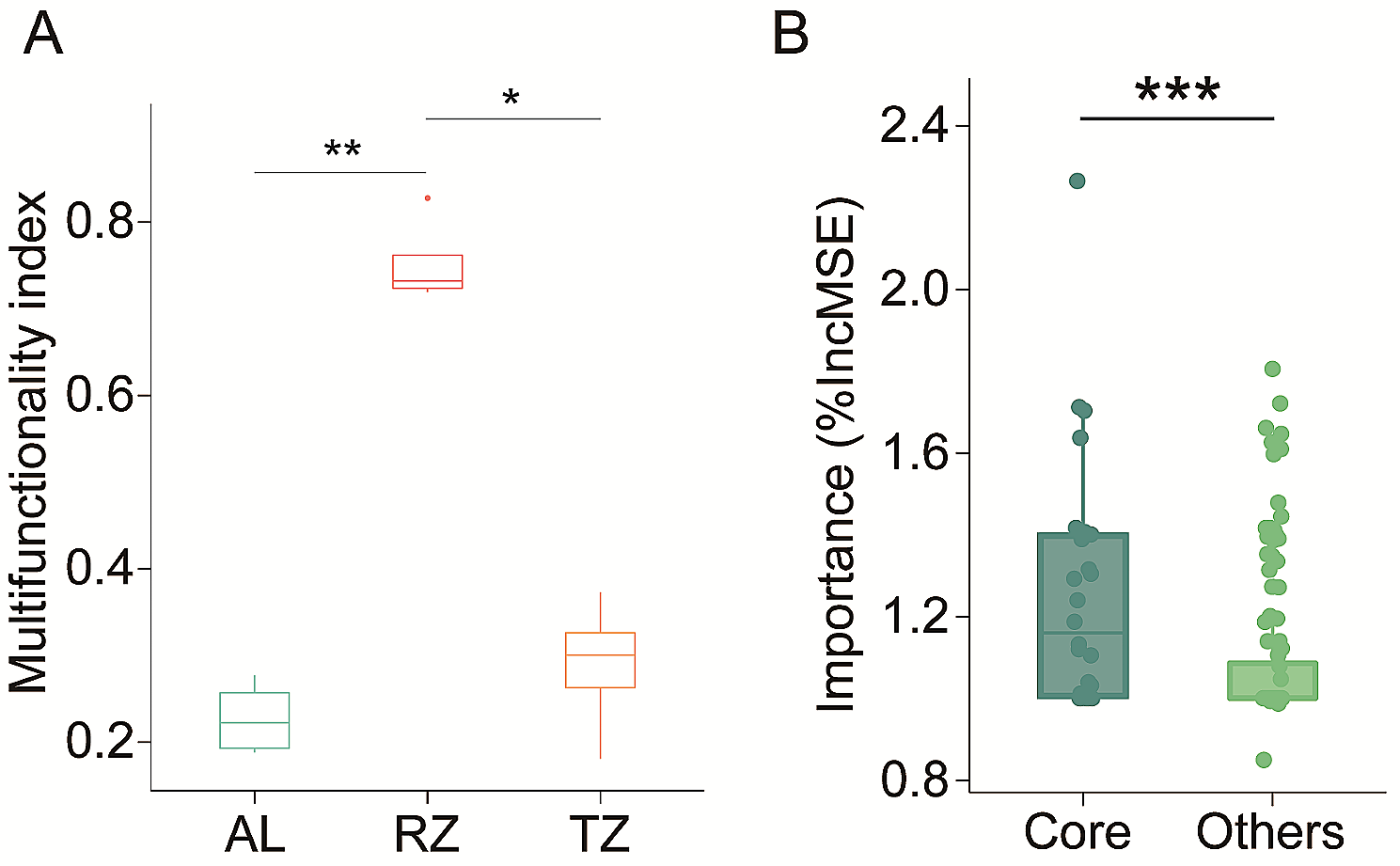
(**A**) Variation in the multi-functionality index in the *C. camphora* coppice planting. Significant differences were found after multiple comparison tests after the Kruskal–Wallis analysis (*p < 0.05; **p < 0.01). (**B**) Random Forest mean predictor importance of microbial taxa for multifunctionality in the *C. camphora* coppice planting. Percentage increases in the MSE (mean squared error) of variables were used to estimate the importance of these predictors, and higher MSE% values implied more important predictors. The boxplot showed differences between core taxa (“Core”) and other non-core taxa (“Others”; ***p < 0.001; Wilcoxon rank-sum test). Note: AL, abandoned land; RZ, root zone; and TZ, transition zone

Compared to the AL, the RZ significantly increased soil TN, AN, and AP contents, and significantly improved soil UE, PPO, and POD activities but decreased soil ACP activity. Specifically, the ecosystem multi-functionality of the soil microbiota increased significantly (by 230.8%) in the RZ compared to the AL (*p* < 0.01). There were also significant differences (*p* < 0.05) between RZ and TZ, and the RZ increased soil ecosystem multi-functionality by 160.8% relative to the TZ. However, no significant difference in ecosystem multi-functionality was observed between AL and TZ (*p* > 0.05; Fig. 4A). RF analysis found the significant influences of core microbiota on soil ecosystem multi-functionality (*p* < 0.001); however, the contribution of the non-core microbiota was less obvious (Fig. 4B).

### The relationship between soil core ecological clusters and soil ecosystem multifunctionality in the *C. camphora* coppice planting

**Fig. 5:**
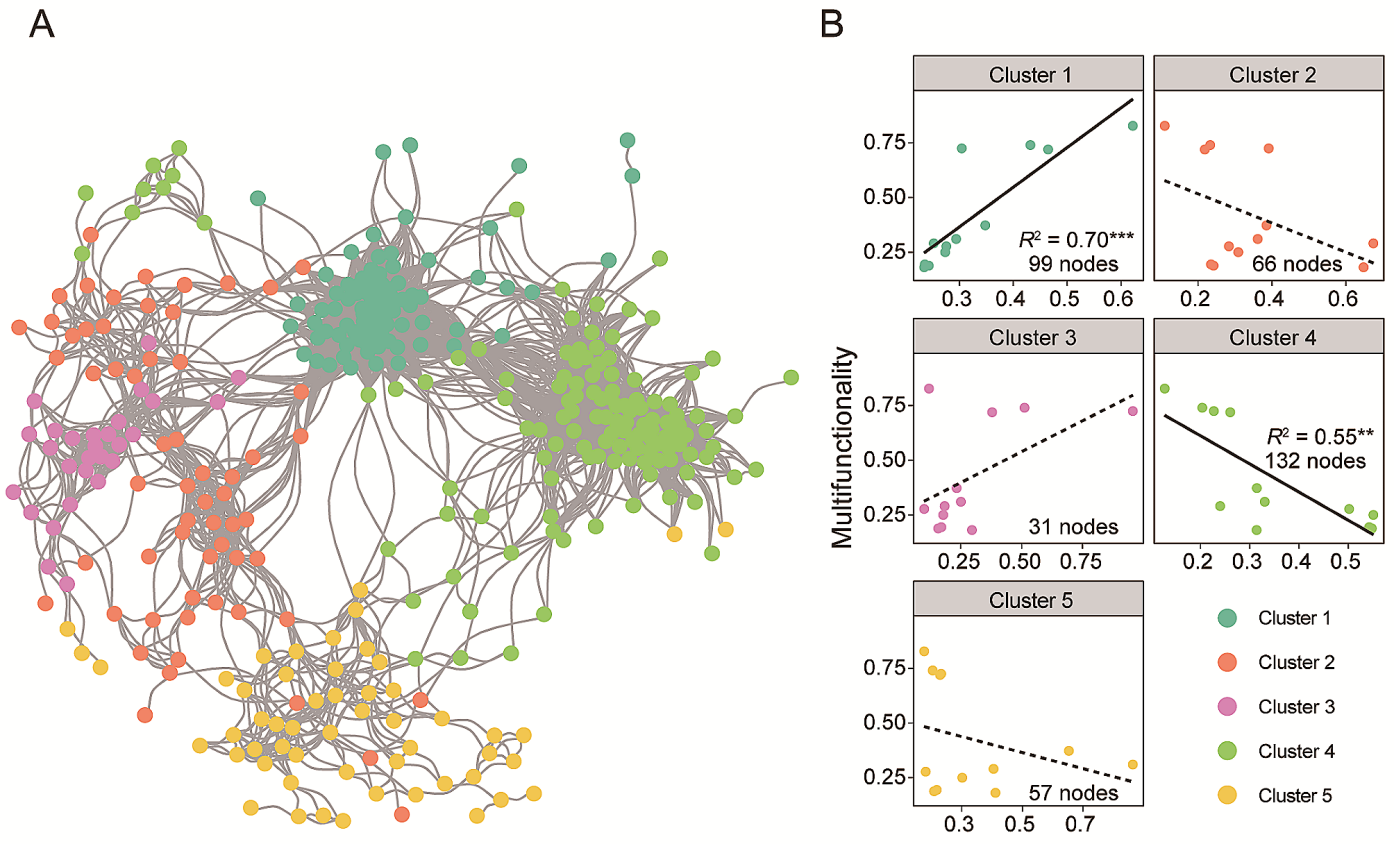
Correlation between the soil core ecological clusters and soil ecosystem multifunctionality. (**A**) Co-occurrence networks based on the Weighted Gene Co-Expression Network Analysis (WGCNA). The colors of the nodes represent different ecological clusters. (**B**) The regression relationships between soil ecological multifunctionality index and the relative abundance of core taxa within the main ecological clusters. Statistical analysis was performed using ordinary least squares linear regressions; *p* values were indicated by asterisks (**p < 0.01; ***p < 0.001)

We utilized a co-occurrence network through WGCNA to explore the soil ecosystem network structure in the *C. camphora* coppice planting. Our findings showed that the network comprised five major ecological clusters with a total of 385 nodes (Fig. 5A). Least square regression analysis showed that cluster 1 had a significantly positive correlation with soil ecosystem multifunctionality (*R*^2^ = 0.70, *p* < 0.001) with 99 nodes. In contrast, there was a significant negative correlation between cluster 4 and soil ecosystem multi-functionality (*R*^2^ = 0.55, *p* < 0.01) with 132 nodes. The remaining clusters had no significant effect on soil ecosystem multi-functionality (*p* > 0.05, Fig. 5B).

### The features of soil core ecological clusters and their relationships

**Fig. 6:**
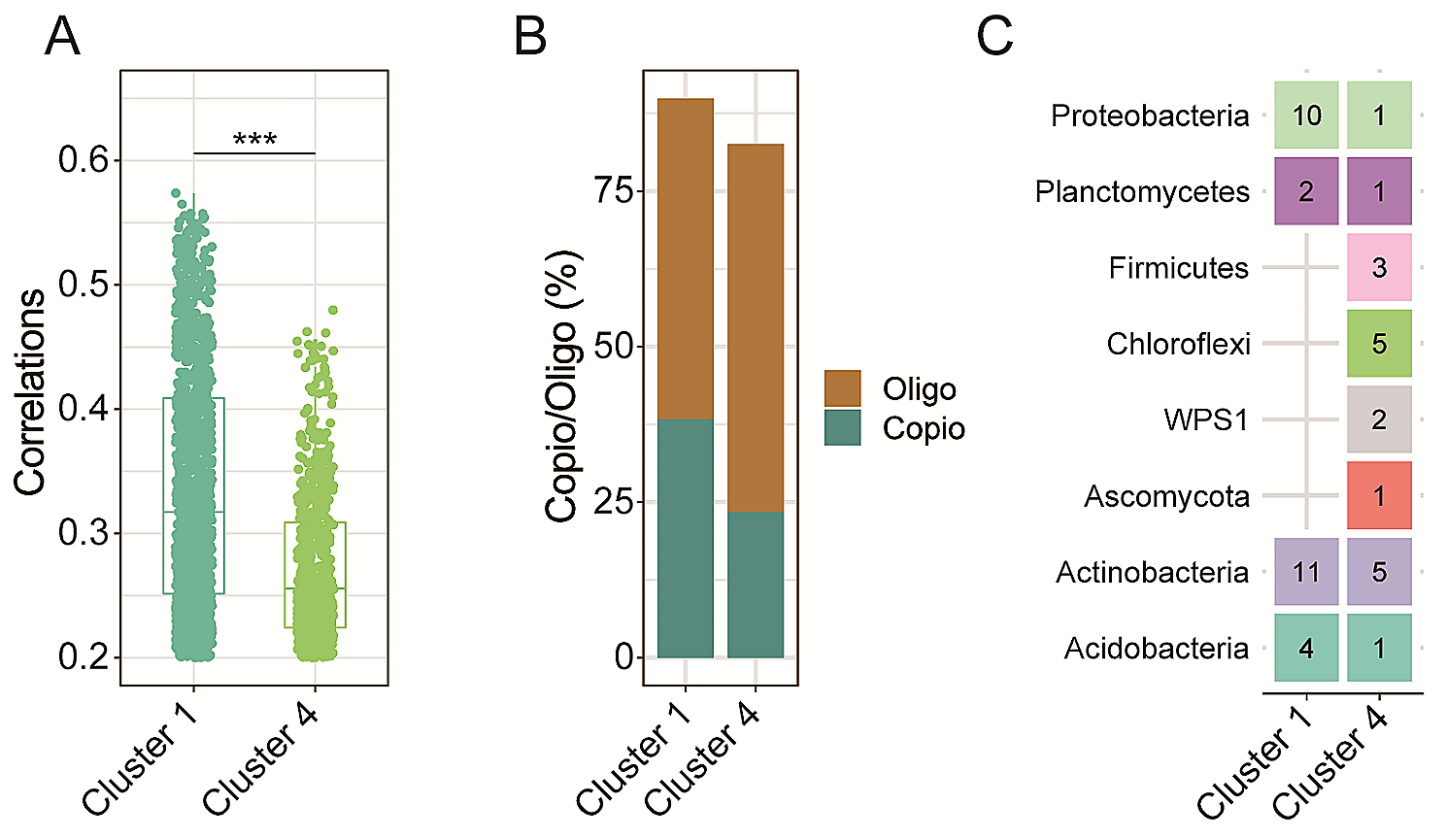
(**A**) Correlations between soil core taxa of cluster 1 and cluster 4 within the ecological network (***p < 0.001). (**B**) Value of *Oligotrophs/Copiotrophs* in cluster 1 and cluster 4. (**C**) The number of soil core microbiota at the phylum level in cluster 1 and cluster 4

The Wilcoxon rank sum test determined the correlations between cluster 1 and cluster 4. We observed that the relevance of the soil core microbiota within cluster 1 was significantly higher than the cluster 4 (*p* < 0.001, Fig. 6A). In cluster 1, *Copiotrophs/ Oligotrophs* (74.50%) were more abundant than in cluster 4 (39.74%) (Fig. 6B). The hub taxa in cluster 1 were dominated by members of Actinobacteria and Proteobacteria, whereas the members of Actinobacteria and Chloroflexi dominated the cluster 4 (Fig. 6C).

**Fig. 7:**
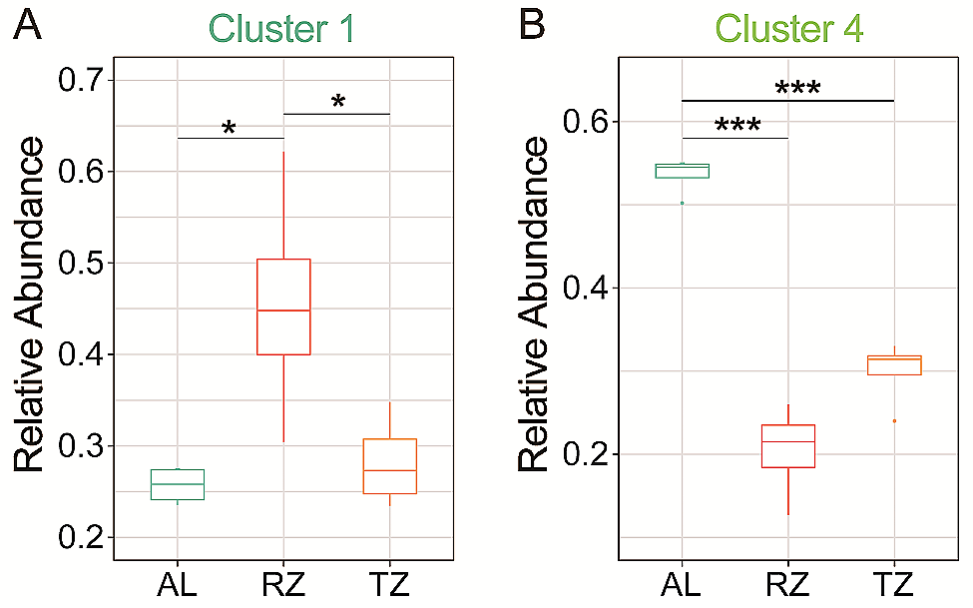
(**A**) The relative abundance of soil core microbiota of cluster 1. (**B**) The relative abundance of soil core microbiota of cluster 4. Note: AL, abandoned land; RZ, root zone; and TZ, transition zone

Regarding the relative abundance of soil key ecological clusters under different sampling points, RZ significantly (*p* < 0.05) increased the relative abundance of cluster 1 compared with AL (by 77.34%) and TZ (by 61.50%) (Fig. 7A). In contrast, AL significantly decreased the relative abundance of cluster 4 by 162.3% compared with RZ and by 78.91% compared with TZ (*p* < 0.01) (Fig. 7B). There was no significant difference between AL and TZ in cluster 1 and RZ and TZ in cluster 4 (Fig. 7).

## Discussion

Anthropogenic pressures have caused ecological deterioration on a global scale, and agricultural ecosystems are experiencing increasing challenges (e.g., species loss) [53, 54]. As a result, it is undoubtedly worth in soil ecosystems’ response pattern to human disturbance and how microbiota dominates ecosystem functional diversity under these changes [55, 56]. Here, we present empirical proof that soil ecosystem multi-functions in our study have improved under *C. camphora* tree planting. Moreover, we found a high contribution of soil core microbiota in the ecological clusters based on the co-occurrence network analysis is of high contribution in maintaining soil multifunctionality. These findings highlight a close connection between soil core microbiota and ecosystem function in the context of *C. camphora* tree planting.

In this study, we found that soil fertility in RZ and TZ was comparatively higher than in the AL, this may be related to the fertilizer application during the *C. camphora* coppice planting. However, many studies have found that even fertilization may not avoid soil fertility decline after land use pattern changes [56–58]. A previous study revealed that the conversion of unfertilized natural forests into tree plantations results in a significant reduction in soil fertility [59]. There are several potential reasons accounting for this. Firstly, although fertilizer can increase soil nutrient input, it is uncertain whether it can compensate for nutrients taken away by crop harvesting [7], specifically the *C. camphora* coppice harvest in this study. Furthermore, soil fertility changes may also correlate with the types of vegetation or crops [4]. For example, tree plantation can provide a more lasting and extensive surface covering relative to abandoned or agricultural land. Thus, it can alleviate soil nutrient loss, especially in the hot and rainy subtropical red soil region [60, 61]. Additionally, different litterfall types and root distribution of plants also have distinct effects on soil fertility [62, 63]. The present study demonstrated that RZ and TZ improved soil fertility compared with abandoned land. This is not only caused by fertilization, but the comprehensive effect of *C. camphora* coppice planting under current planting management patterns.

Tree planting positively impacts the soil ecosystem by improving soil structure, regulating nutrient cycling [64]. Our results showed a significant increase in soil multi-functionality in the RZ under *C. camphora* coppice planting (*p* < 0.01) compared to the abandoned land (Fig. 4A), implying the significance of tree planting in regulating ecosystem multi-functionality. Consistent with our findings, Yan et al. [55] demonstrated that the *Robinia pseudoacacia* tree planting is conducive to maintaining soil ecosystem multi-functionality in northern China. These findings suggest that the nutrients accumulation from leaf litter and other dead materials overtime increases soil organic matter, which positively affects overall soil health and functions [63, 65]. Moreover, trees and the expansion of their root systems in the soil provide an important source of nutrients to microbiota through root exudates that create a diverse niche for a range of microbiota, supporting biodiverse and healthy soil [66]. Collectively, tree planting and healthy soils can improve ecosystem resilience to natural and anthropogenic environmental changes while sustaining their multifunctionality [67].

Soil microbiota have been identified as the most active component of the soil ecosystem [68]. The diversity, composition, and activities of these microbiota interact with various factors in the soil to regulate multiple critical ecological functions [69]. Through empirical research, we discovered notable distinctions in the community composition of soil core microbiota and variations in their ecological preferences within the *C. camphora* coppice planting. For example, in the RZ, the most commonly found taxa were Proteobacteria, which are known to thrive in high-nutrient environments due to their copiotrophic nature and ability to grow rapidly [70]. However, in the AL, Chloroflexi was the most abundant taxa and the microorganisms in the taxa are oligotrophic that generate energy through photosynthesis and break down organic matter in low-nutrient conditions [71]. By studying the functional roles of various microbial taxa, we can gain valuable insights into their contributions to crucial ecological processes such as nutrient cycling and carbon sequestration. However, further experimental and modeling approaches exploring the variation in microbiota composition and structure and the underlying mechanisms of such variations are needed to understand the long-term impacts of microbiota on soil ecosystem dynamics.

Some important soil functions are primarily determined by specific soil microbial taxa, rather than the entire microbial community [19]. Recently, researchers have focused on soil microbiota (soil core taxa, dominant taxa, or rare taxa) and ecosystem multi-functionality [43, 72, 73]. These studies highlight the importance of soil core microbiota influencing the multiple functions in soil ecosystems [74]. For example, Jiao et al. [11] revealed positive relationships between soil core taxa and ecosystem functions (e.g., nutrient cycling). Consistently, we reported that the soil core microbiota under the *C. camphora* coppice planting maintained significantly higher multiple ecosystem functions than the non-core microbiota (*p* < 0.001; Fig. 4B). This underscores the crucial role of core microbiota in preserving the multifaceted functions of soil ecosystems [75]. Notably, core microbiota are more generalist in distribution, where they can utilize a broad range of niches and resources, enhancing the productivity and stability of the soil ecosystems [76, 77].

Our results showed that the core taxa across the sampling points formed distinct ecological clusters (Fig. 5A), suggesting that the connections among these core taxa in the network were tied to their resource preferences. Such clustering due to resource preference and interaction between taxa within the cluster enhances the ecosystem multi-functionality [26]. Indeed, our research demonstrated that there was a strong positive association between the relative abundance of ecological cluster 1 and ecosystem multifunctionality (Fig. 5B), suggesting that the particular soil microbiota with comparable resource preferences play a crucial role in supporting various soil functions. The observed relationships can be explained by the following two factors. First, the tightness of microbial networks can enhance nutrient uptake and utilization [78]. In fact, soil core taxa within cluster 1 showed a strong connection to ecological multi-functionality. Therefore, higher co-occurrence and tightness of soil core taxa in ecological cluster 1 may have enhanced soil functions, including improved nutrient cycling [79]. Second, soil core microbiota with different ecological strategies can also impact the links between ecological clusters and multi-functionality [80, 81]. Specifically, we found a higher *Copiotrophs/Oligotrophs* ratio and increased relative abundance of copiotrophic taxa in cluster 1 may coincide with the improvement of soil ecological functions, such as organic matter, as copiotrophic species are capable of utilizing a wide variety of nutrients [82, 83].

## Conclusion

In conclusion, our results showed that the *C. camphora* coppice planting altered the community composition of soil core microbiota and improved the overall functionality of soil ecosystems. In addition, our findings highlight the significance of soil core microbiota in maintaining the functionality of agroecosystems, such as the *C. camphora* coppice planting. Notably, the relative abundance of soil core microbiota within key ecological clusters significantly impacted the soil’s ability to perform multiple functions, suggesting that specific microbiota are crucial to maintaining soil functions in the *C. camphora* coppice planting. Therefore, maintaining diverse soil microbiota through tree planting may be the key to conserving soil health and maintaining agricultural sustainability across agricultural landscapes undergoing rapid anthropogenic changes.

### Electronic supplementary material

Below is the link to the electronic supplementary material.

Supplementary Material 1

## Data Availability

The datasets for this study can be found in Sequence Read Archive (SRP390129).

## Abbreviations

*C. camphora*: *Cinnamomum camphora* (L.) Presl
SOC: Soil organic carbon
TN: Total nitrogen
AN: Available nitrogen
AP: Available phosphorus
INV: Invertase
UE: Urease
ACP: Acid phosphatase
CAT: Catalase
PPO: Polyphenol oxidase
POD: Peroxidase
