# Supplementary material for: Core microbiota drive multi-functionality of the soil microbiome in the Cinnamomum camphora coppice planting
Source: BMC Microbiol. 2024 Jan 10;24:18. doi: 10.1186/s12866-023-03170-8 (PMC10777636; doi:10.1186/s12866-023-03170-8)
Supplement: Supplementary file 1 — Supplementary Material 1 [file 12866_2023_3170_MOESM1_ESM.docx]

Table S1 Distribution of soil core microbiota at different taxonomy levels

| ID | module | kingdom | phylum | class | order | family | genus |
| --- | --- | --- | --- | --- | --- | --- | --- |
| OTU_3 | turquoise | K_Bacteria | p_Proteobacteria | c_Alphaproteobacteria | o_Rhizobiales | f_Bradyrhizobiaceae | g_Bradyrhizobium |
| OTU_2 | brown | K_Bacteria | p_Proteobacteria | c_Gammaproteobacteria | o_Xanthomonadales | f_Rhodanobacteraceae | g_Rhodanobacter |
| OTU_1 | green | K_Bacteria | p_Actinobacteria | c_Actinobacteria | o_Micrococcales | f_Micrococcaceae | g_Arthrobacter |
| OTU_5 | turquoise | K_Bacteria | p_Proteobacteria | c_Alphaproteobacteria | o_Rhizobiales | f_Bradyrhizobiaceae | g_Pseudorhodoplanes |
| OTU_16 | blue | K_Bacteria | p_Acidobacteria | c_Acidobacteriae | o_Gp1 | f_Gp1 | g_Gp1 |
| OTU_4 | green | K_Bacteria | p_Proteobacteria | c_Gammaproteobacteria | o_Xanthomonadales | f_Xanthomonadaceae | g_Lysobacter |
| OTU_30 | blue | K_Bacteria | p_Acidobacteria | c_Acidobacteriae | o_Gp1 | f_Gp1 | g_Gp1 |
| OTU_28 | green | K_Bacteria | p_Proteobacteria | c_Gammaproteobacteria | o_Xanthomonadales | f_Xanthomonadaceae | g_Thermomonas |
| OTU_19 | brown | K_Bacteria | p_Acidobacteria | c_Acidobacteriae | o_Gp2 | f_Gp2 | g_Gp2 |
| OTU_6 | yellow | K_Bacteria | p_Actinobacteria | c_Actinobacteria | o_Pseudonocardiales | f_Pseudonocardiaceae | g_Pseudonocardia |
| OTU_31 | turquoise | K_Bacteria | p_Acidobacteria | c_Acidobacteriae | o_Occallatibacter | f_Occallatibacter | g_Occallatibacter |
| OTU_22 | yellow | K_Bacteria | p_Bacteroidetes | c_Cytophagia | o_Cytophagales | f_Fulvivirgaceae | g_Chryseolinea |
| OTU_155 | blue | K_Bacteria | p_Acidobacteria | c_Acidobacteriae | o_Gp6 | f_Gp6 | g_Gp6 |
| OTU_33 | turquoise | K_Bacteria | p_Proteobacteria | c_Gammaproteobacteria | o_Gammaproteobacteria_incertae_sedis | f_Acidibacter | g_Acidibacter |
| OTU_32 | turquoise | K_Bacteria | p_Chloroflexi | c_Ktedonobacteria | o_Ktedonobacterales | f_Ktedonobacteraceae | g_Ktedonobacter |
| OTU_12 | yellow | K_Bacteria | p_Bacteroidetes | c_Chitinophagia | o_Chitinophagales | f_Chitinophagaceae | g_Flavitalea |
| OTU_11 | turquoise | K_Bacteria | p_Chloroflexi | c_Ktedonobacteria | o_Ktedonobacterales | f_Ktedonobacteraceae | g_Ktedonobacter |
| OTU_39 | yellow | K_Bacteria | p_Proteobacteria | c_Betaproteobacteria | o_Burkholderiales | f_Comamonadaceae | g_Variovorax |
| OTU_36 | blue | K_Bacteria | p_Acidobacteria | c_Acidobacteriae | o_Gp1 | f_Gp1 | g_Gp1 |
| OTU_1143 | turquoise | K_Bacteria | p_Proteobacteria | c_Alphaproteobacteria | o_Rhizobiales | f_Bradyrhizobiaceae | g_Pseudorhodoplanes |
| OTU_15 | brown | K_Bacteria | p_Bacteroidetes | c_Sphingobacteriia | o_Sphingobacteriales | f_Sphingobacteriaceae | g_Mucilaginibacter |
| OTU_137 | blue | K_Bacteria | p_Acidobacteria | c_Acidobacteriae | o_Gp6 | f_Gp6 | g_Gp6 |
| OTU_91 | blue | K_Bacteria | p_Proteobacteria | c_Alphaproteobacteria | o_Rhodospirillales | f_Rhodospirillaceae | g_Aliidongia |
| OTU_29 | turquoise | K_Bacteria | p_Actinobacteria | c_Actinobacteria | o_Streptomycetales | f_Streptomycetaceae | g_Streptacidiphilus |
| OTU_93 | turquoise | K_Bacteria | p_Actinobacteria | c_Actinobacteria | o_Streptosporangiales | f_Thermomonosporaceae | g_Spirillospora |
| OTU_96 | yellow | K_Bacteria | p_Acidobacteria | c_Acidobacteriae | o_Candidatus Solibacter | f_Candidatus Solibacter | g_Candidatus Solibacter |
| OTU_13 | turquoise | K_Bacteria | p_Chloroflexi | c_Ktedonobacteria | o_Ktedonobacterales | f_Ktedonobacteraceae | g_Ktedonobacter |
| OTU_23 | turquoise | K_Bacteria | p_Actinobacteria | c_Actinobacteria | o_Streptosporangiales | f_Thermomonosporaceae | g_Actinoallomurus |
| OTU_17 | turquoise | K_Bacteria | p_Proteobacteria | c_Alphaproteobacteria | o_Sphingomonadales | f_Sphingomonadaceae | g_Sphingomonas |
| OTU_60 | brown | K_Bacteria | p_Proteobacteria | c_Gammaproteobacteria | o_Gammaproteobacteria_incertae_sedis | f_Acidibacter | g_Acidibacter |
| OTU_35 | brown | K_Bacteria | p_Proteobacteria | c_Betaproteobacteria | o_Burkholderiales | f_Burkholderiaceae | g_Paraburkholderia |
| OTU_26 | green | K_Bacteria | p_Acidobacteria | c_Acidobacteriae | o_Gp3 | f_Gp3 | g_Gp3 |
| OTU_775 | brown | K_Bacteria | p_Proteobacteria | c_Gammaproteobacteria | o_Xanthomonadales | f_Rhodanobacteraceae | g_Rudaea |
| OTU_99 | yellow | K_Bacteria | p_Acidobacteria | c_Acidobacteriae | o_Gp3 | f_Gp3 | g_Gp3 |
| OTU_48 | yellow | K_Bacteria | p_Acidobacteria | c_Acidobacteriae | o_Gp1 | f_Gp1 | g_Gp1 |
| OTU_7 | blue | K_Bacteria | p_Proteobacteria | c_Betaproteobacteria | o_Burkholderiales | f_Comamonadaceae | g_Aquincola |
| OTU_20 | blue | K_Bacteria | p_Actinobacteria | c_Actinobacteria | o_Mycobacteriales | f_Nocardiaceae | g_Rhodococcus |
| OTU_79 | turquoise | K_Bacteria | p_Chloroflexi | c_Anaerolineae | o_Anaerolineales | f_Anaerolineaceae | g_Thermomarinilinea |
| OTU_55 | blue | K_Bacteria | p_Proteobacteria | c_Alphaproteobacteria | o_Rhizobiales | f_Bradyrhizobiaceae | g_Pseudolabrys |
| OTU_53 | turquoise | K_Bacteria | p_Firmicutes | c_Clostridia | o_Thermosediminibacterales | f_Thermosediminibacteraceae | g_Fervidicola |
| OTU_10 | turquoise | K_Bacteria | p_Proteobacteria | c_Alphaproteobacteria | o_Rhizobiales | f_Phyllobacteriaceae | g_Mesorhizobium |
| OTU_117 | blue | K_Bacteria | p_Acidobacteria | c_Acidobacteriae | o_Edaphobacter | f_Edaphobacter | g_Edaphobacter |
| OTU_110 | turquoise | K_Bacteria | p_Actinobacteria | c_Actinobacteria | o_Mycobacteriales | f_Mycobacteriaceae | g_Mycobacterium |
| OTU_74 | brown | K_Bacteria | p_Acidobacteria | c_Acidobacteriae | o_Gp2 | f_Gp2 | g_Gp2 |
| OTU_444 | yellow | K_Bacteria | p_Proteobacteria | c_Alphaproteobacteria | o_Rhizobiales | f_Bradyrhizobiaceae | g_Pseudorhodoplanes |
| OTU_150 | turquoise | K_Bacteria | p_Firmicutes | c_Bacilli | o_Bacillales | f_Bacillaceae 1 | g_Neobacillus |
| OTU_83 | brown | K_Bacteria | p_Acidobacteria | c_Acidobacteriae | o_Gp3 | f_Gp3 | g_Gp3 |
| OTU_49 | brown | K_Bacteria | p_Proteobacteria | c_Alphaproteobacteria | o_Rhodospirillales | f_Azospirillaceae | g_Skermanella |
| OTU_44 | yellow | K_Bacteria | p_Acidobacteria | c_Acidobacteriae | o_Gp2 | f_Gp2 | g_Gp2 |
| OTU_45 | turquoise | K_Bacteria | p_Acidobacteria | c_Acidobacteriae | o_Edaphobacter | f_Edaphobacter | g_Edaphobacter |
| OTU_24 | brown | K_Bacteria | p_Proteobacteria | c_Deltaproteobacteria | o_Desulfuromonadales | f_Geobacteraceae | g_Geothermobacter |
| OTU_106 | brown | K_Bacteria | p_Acidobacteria | c_Acidobacteriae | o_Gp2 | f_Gp2 | g_Gp2 |
| OTU_72 | turquoise | K_Bacteria | p_Actinobacteria | c_Thermoleophilia | o_Solirubrobacterales | f_Conexibacteraceae | g_Conexibacter |
| OTU_75 | blue | K_Bacteria | p_Proteobacteria | c_Alphaproteobacteria | o_Rhizobiales | f_Hyphomicrobiaceae | g_Pedomicrobium |
| OTU_105 | blue | K_Bacteria | p_Acidobacteria | c_Acidobacteriae | o_Paludibaculum | f_Paludibaculum | g_Paludibaculum |
| OTU_1956 | turquoise | K_Bacteria | p_Actinobacteria | c_Thermoleophilia | o_Solirubrobacterales | f_Conexibacteraceae | g_Conexibacter |
| OTU_2818 | blue | K_Bacteria | p_Proteobacteria | c_Alphaproteobacteria | o_Rhizobiales | f_Bradyrhizobiaceae | g_Afipia |
| OTU_89 | brown | K_Bacteria | p_Acidobacteria | c_Acidobacteriae | o_Gp1 | f_Gp1 | g_Gp1 |
| OTU_54 | green | K_Bacteria | p_Proteobacteria | c_Betaproteobacteria | o_Burkholderiales | f_Comamonadaceae | g_Rhizobacter |
| OTU_25 | turquoise | K_Bacteria | p_Actinobacteria | c_Actinobacteria | o_Micrococcales | f_Intrasporangiaceae | g_Pedococcus |
| OTU_90 | turquoise | K_Bacteria | p_Actinobacteria | c_Thermoleophilia | o_Solirubrobacterales | f_Conexibacteraceae | g_Conexibacter |
| OTU_241 | turquoise | K_Bacteria | p_Chloroflexi | c_Ktedonobacteria | o_Ktedonobacterales | f_Ktedonobacteraceae | g_Ktedonobacter |
| OTU_175 | brown | K_Bacteria | p_Proteobacteria | c_Betaproteobacteria | o_Burkholderiales | f_Burkholderiaceae | g_Caballeronia |
| OTU_9 | turquoise | K_Bacteria | p_Actinobacteria | c_Actinobacteria | o_Mycobacteriales | f_Nocardiaceae | g_Rhodococcus |
| OTU_71 | brown | K_Bacteria | p_Proteobacteria | c_Alphaproteobacteria | o_Rhodospirillales | f_Azospirillaceae | g_Nitrospirillum |
| OTU_8 | turquoise | K_Bacteria | p_Proteobacteria | c_Betaproteobacteria | o_Burkholderiales | f_Comamonadaceae | g_Rhodoferax |
| OTU_3061 | blue | K_Bacteria | p_Proteobacteria | c_Alphaproteobacteria | o_Rhizobiales | f_Bradyrhizobiaceae | g_Pseudolabrys |
| OTU_1995 | yellow | K_Bacteria | p_Acidobacteria | c_Acidobacteriae | o_Gp1 | f_Gp1 | g_Gp1 |
| OTU_21 | turquoise | K_Bacteria | p_Chloroflexi | c_Thermomicrobia | o_Sphaerobacterales | f_Sphaerobacteraceae | g_Sphaerobacter |
| OTU_127 | blue | K_Bacteria | p_Proteobacteria | c_Alphaproteobacteria | o_Rhodospirillales | f_Rhodospirillaceae | g_Dongia |
| OTU_81 | turquoise | K_Bacteria | p_Proteobacteria | c_Alphaproteobacteria | o_Rhodospirillales | f_Acetobacteraceae | g_Acidisoma |
| OTU_14 | brown | K_Bacteria | p_Proteobacteria | c_Alphaproteobacteria | o_Caulobacterales | f_Caulobacteraceae | g_Phenylobacterium |
| OTU_107 | blue | K_Bacteria | p_Proteobacteria | c_Alphaproteobacteria | o_Rhizobiales | f_Blastochloridaceae | g_Blastochloris |
| OTU_34 | turquoise | K_Bacteria | p_Chloroflexi | c_Ktedonobacteria | o_Ktedonobacterales | f_Ktedonobacteraceae | g_Ktedonobacter |
| OTU_116 | blue | K_Bacteria | p_Actinobacteria | c_Actinobacteria | o_Streptomycetales | f_Streptomycetaceae | g_Streptomyces |
| OTU_50 | blue | K_Bacteria | p_Proteobacteria | c_Alphaproteobacteria | o_Rhodospirillales | f_Rhodospirillaceae | g_Lacibacterium |
| OTU_58 | turquoise | K_Bacteria | p_Proteobacteria | c_Alphaproteobacteria | o_Rhizobiales | f_Bradyrhizobiaceae | g_Pseudorhodoplanes |
| OTU_27 | turquoise | K_Bacteria | p_Firmicutes | c_Clostridia | o_Clostridiales | f_Symbiobacteriaceae | g_Caldinitratiruptor |
| OTU_122 | yellow | K_Bacteria | p_Planctomycetes | c_Planctomycetacia | o_Planctomycetales | f_Planctomycetaceae | g_Rubinisphaera |
| OTU_184 | blue | K_Bacteria | p_Proteobacteria | c_Alphaproteobacteria | o_Caulobacterales | f_Caulobacteraceae | g_Phenylobacterium |
| OTU_98 | brown | K_Bacteria | p_Acidobacteria | c_Acidobacteriae | o_Gp2 | f_Gp2 | g_Gp2 |
| OTU_1313 | blue | K_Bacteria | p_Proteobacteria | c_Alphaproteobacteria | o_Rhizobiales | f_Bradyrhizobiaceae | g_Pseudorhodoplanes |
| OTU_124 | brown | K_Bacteria | p_Verrucomicrobia | c_Subdivision3 | o_Limisphaera | f_Limisphaera | g_Limisphaera |
| OTU_64 | brown | K_Bacteria | p_Acidobacteria | c_Acidobacteriae | o_Gp2 | f_Gp2 | g_Gp2 |
| OTU_97 | turquoise | K_Bacteria | p_Proteobacteria | c_Gammaproteobacteria | o_Gammaproteobacteria_incertae_sedis | f_Acidibacter | g_Acidibacter |
| OTU_216 | yellow | K_Bacteria | p_Acidobacteria | c_Acidobacteriae | o_Gp1 | f_Gp1 | g_Gp1 |
| OTU_3875 | yellow | K_Bacteria | p_Acidobacteria | c_Acidobacteriae | o_Gp2 | f_Gp2 | g_Gp2 |
| OTU_56 | green | K_Bacteria | p_Proteobacteria | c_Alphaproteobacteria | o_Sphingomonadales | f_Erythrobacteraceae | g_Novosphingobium |
| OTU_82 | blue | K_Bacteria | p_Chloroflexi | c_Ktedonobacteria | o_Ktedonobacterales | f_Ktedonobacteraceae | g_Ktedonobacter |
| OTU_246 | blue | K_Bacteria | p_Proteobacteria | c_Betaproteobacteria | o_Burkholderiales | f_Comamonadaceae | g_Tepidimonas |
| OTU_43 | turquoise | K_Bacteria | p_Actinobacteria | c_Actinobacteria | o_Micrococcales | f_Micrococcaceae | g_Sinomonas |
| OTU_195 | brown | K_Bacteria | p_Acidobacteria | c_Acidobacteriae | o_Gp3 | f_Gp3 | g_Gp3 |
| OTU_685 | blue | K_Bacteria | p_Actinobacteria | c_Thermoleophilia | o_Gaiellales | f_Gaiellaceae | g_Gaiella |
| OTU_235 | blue | K_Bacteria | p_Actinobacteria | c_Thermoleophilia | o_Solirubrobacterales | f_Baekduiaceae | g_Baekduia |
| OTU_84 | yellow | K_Bacteria | p_Proteobacteria | c_Alphaproteobacteria | o_Rhodospirillales | f_Acetobacteraceae | g_Acidisoma |
| OTU_245 | turquoise | K_Bacteria | p_Actinobacteria | c_Actinobacteria | o_Streptosporangiales | f_Thermomonosporaceae | g_Actinoallomurus |
| OTU_102 | blue | K_Bacteria | p_Proteobacteria | c_Betaproteobacteria | o_Burkholderiales | f_Oxalobacteraceae | g_Massilia |
| OTU_66 | green | K_Bacteria | p_Proteobacteria | c_Betaproteobacteria | o_Nitrosomonadales | f_Methylophilaceae | g_Methylobacillus |
| OTU_133 | yellow | K_Bacteria | p_Acidobacteria | c_Acidobacteriae | o_Gp1 | f_Gp1 | g_Gp1 |
| OTU_172 | yellow | K_Bacteria | p_Proteobacteria | c_Alphaproteobacteria | o_Rhizobiales | f_Roseiarcaceae | g_Roseiarcus |
| OTU_394 | blue | K_Bacteria | p_Actinobacteria | c_Thermoleophilia | o_Gaiellales | f_Gaiellaceae | g_Gaiella |
| OTU_585 | turquoise | K_Bacteria | p_Acidobacteria | c_Acidobacteriae | o_Gp2 | f_Gp2 | g_Gp2 |
| OTU_92 | green | K_Bacteria | p_Acidobacteria | c_Acidobacteriae | o_Gp3 | f_Gp3 | g_Gp3 |
| OTU_40 | turquoise | K_Bacteria | p_Actinobacteria | c_Actinobacteria | o_Propionibacteriales | f_Nocardioidaceae | g_Marmoricola |
| OTU_136 | blue | K_Bacteria | p_Acidobacteria | c_Acidobacteriae | o_Gp16 | f_Gp16 | g_Gp16 |
| OTU_57 | turquoise | K_Bacteria | p_Proteobacteria | c_Alphaproteobacteria | o_Rhodospirillales | f_Acetobacteraceae | g_Acidisoma |
| OTU_37 | yellow | K_Bacteria | p_Proteobacteria | c_Deltaproteobacteria | o_Myxococcales | f_Labilitrichaceae | g_Labilithrix |
| OTU_193 | green | K_Bacteria | p_Acidobacteria | c_Acidobacteriae | o_Gp3 | f_Gp3 | g_Gp3 |
| OTU_459 | turquoise | K_Bacteria | p_Proteobacteria | c_Alphaproteobacteria | o_Micropepsales | f_Micropepsaceae | g_Micropepsis |
| OTU_758 | brown | K_Bacteria | p_Acidobacteria | c_Acidobacteriae | o_Gp2 | f_Gp2 | g_Gp2 |
| OTU_223 | blue | K_Bacteria | p_Planctomycetes | c_Planctomycetacia | o_Pirellulales | f_Thermoguttaceae | g_Thermostilla |
| OTU_42 | turquoise | K_Bacteria | p_Chloroflexi | c_Ktedonobacteria | o_Ktedonobacterales | f_Ktedonobacteraceae | g_Ktedonobacter |
| OTU_168 | blue | K_Bacteria | p_Proteobacteria | c_Gammaproteobacteria | o_Pseudomonadales | f_Pseudomonadaceae | g_Pseudomonas |
| OTU_1127 | turquoise | K_Bacteria | p_Acidobacteria | c_Acidobacteriae | o_Gp1 | f_Gp1 | g_Gp1 |
| OTU_250 | yellow | K_Bacteria | p_Proteobacteria | c_Alphaproteobacteria | o_Micropepsales | f_Micropepsaceae | g_Micropepsis |
| OTU_165 | blue | K_Bacteria | p_Chloroflexi | c_Ktedonobacteria | o_Ktedonobacterales | f_Thermosporotrichaceae | g_Thermosporothrix |
| OTU_2007 | turquoise | K_Bacteria | p_Actinobacteria | c_Actinobacteria | o_Streptosporangiales | f_Thermomonosporaceae | g_Spirillospora |
| OTU_189 | green | K_Bacteria | p_Proteobacteria | c_Alphaproteobacteria | o_Caulobacterales | f_Caulobacteraceae | g_Phenylobacterium |
| OTU_322 | yellow | K_Bacteria | p_Bacteroidetes | c_Chitinophagia | o_Chitinophagales | f_Chitinophagaceae | g_Flavitalea |
| OTU_151 | turquoise | K_Bacteria | p_Proteobacteria | c_Gammaproteobacteria | o_Gammaproteobacteria_incertae_sedis | f_Acidibacter | g_Acidibacter |
| OTU_153 | turquoise | K_Bacteria | p_Chloroflexi | c_Ktedonobacteria | o_Ktedonobacterales | f_Ktedonobacteraceae | g_Ktedonobacter |
| OTU_4419 | yellow | K_Bacteria | p_Acidobacteria | c_Acidobacteriae | o_Silvibacterium | f_Silvibacterium | g_Silvibacterium |
| OTU_108 | blue | K_Bacteria | p_Planctomycetes | c_Planctomycetacia | o_Planctomycetales | f_Gemmataceae | g_Gemmata |
| OTU_112 | yellow | K_Bacteria | p_Actinobacteria | c_Acidimicrobiia | o_Acidimicrobiales | f_Iamiaceae | g_Iamia |
| OTU_601 | blue | K_Bacteria | p_Actinobacteria | c_Actinobacteria | o_Micromonosporales | f_Micromonosporaceae | g_Salinispora |
| OTU_2932 | turquoise | K_Bacteria | p_Proteobacteria | c_Gammaproteobacteria | o_Gammaproteobacteria_incertae_sedis | f_Acidibacter | g_Acidibacter |
| OTU_115 | turquoise | K_Bacteria | p_Firmicutes | c_Clostridia | o_Thermosediminibacterales | f_Tepidanaerobacteraceae | g_Biomaibacter |
| OTU_47 | turquoise | K_Bacteria | p_Actinobacteria | c_Actinobacteria | o_Propionibacteriales | f_Nocardioidaceae | g_Nocardioides |
| OTU_487 | turquoise | K_Bacteria | p_Acidobacteria | c_Acidobacteriae | o_Gp2 | f_Gp2 | g_Gp2 |
| OTU_101 | green | K_Bacteria | p_Verrucomicrobia | c_Opitutae | o_Opitutales | f_Opitutaceae | g_Opitutus |
| OTU_221 | brown | K_Bacteria | p_Proteobacteria | c_Deltaproteobacteria | o_Desulfuromonadales | f_Geobacteraceae | g_Geothermobacter |
| OTU_178 | yellow | K_Bacteria | p_Chloroflexi | c_Ktedonobacteria | o_Ktedonobacterales | f_Ktedonobacteraceae | g_Ktedonobacter |
| OTU_761 | brown | K_Bacteria | p_Proteobacteria | c_Alphaproteobacteria | o_Rhodospirillales | f_Reyranellaceae | g_Reyranella |
| OTU_253 | yellow | K_Bacteria | p_Chloroflexi | c_Ktedonobacteria | o_Ktedonobacterales | f_Ktedonobacteraceae | g_Ktedonobacter |
| OTU_142 | blue | K_Bacteria | p_Proteobacteria | c_Alphaproteobacteria | o_Rhizobiales | f_Bradyrhizobiaceae | g_Variibacter |
| OTU_114 | brown | K_Bacteria | p_Bacteroidetes | c_Sphingobacteriia | o_Sphingobacteriales | f_Sphingobacteriaceae | g_Mucilaginibacter |
| OTU_257 | brown | K_Bacteria | p_Acidobacteria | c_Acidobacteriae | o_Gp1 | f_Gp1 | g_Gp1 |
| OTU_65 | turquoise | K_Bacteria | p_Chloroflexi | c_Ktedonobacteria | o_Ktedonobacterales | f_Ktedonobacteraceae | g_Ktedonobacter |
| OTU_212 | green | K_Bacteria | p_Proteobacteria | c_Deltaproteobacteria | o_Myxococcales | f_Labilitrichaceae | g_Labilithrix |
| OTU_419 | blue | K_Bacteria | p_Actinobacteria | c_Thermoleophilia | o_Solirubrobacterales | f_Baekduiaceae | g_Baekduia |
| OTU_306 | brown | K_Bacteria | p_Proteobacteria | c_Deltaproteobacteria | o_Myxococcales | f_Labilitrichaceae | g_Labilithrix |
| OTU_1356 | blue | K_Bacteria | p_Acidobacteria | c_Acidobacteriae | o_Gp6 | f_Gp6 | g_Gp6 |
| OTU_167 | blue | K_Bacteria | p_Gemmatimonadetes | c_Gemmatimonadetes | o_Gemmatimonadales | f_Gemmatimonadaceae | g_Roseisolibacter |
| OTU_129 | brown | K_Bacteria | p_Chloroflexi | c_Ktedonobacteria | o_Ktedonobacterales | f_Ktedonobacteraceae | g_Ktedonobacter |
| OTU_38 | turquoise | K_Bacteria | p_Proteobacteria | c_Betaproteobacteria | o_Burkholderiales | f_Oxalobacteraceae | g_Glaciimonas |
| OTU_2765 | green | K_Bacteria | p_Acidobacteria | c_Acidobacteriae | o_Gp3 | f_Gp3 | g_Gp3 |
| OTU_94 | brown | K_Bacteria | p_Proteobacteria | c_Gammaproteobacteria | o_Gammaproteobacteria_incertae_sedis | f_Acidibacter | g_Acidibacter |
| OTU_103 | blue | K_Bacteria | p_Firmicutes | c_Clostridia | o_Thermoanaerobacterales | f_Thermoanaerobacteraceae | g_Syntrophaceticus |
| OTU_158 | turquoise | K_Bacteria | p_Actinobacteria | c_Actinobacteria | o_Catenulisporales | f_Actinospicaceae | g_Actinocrinis |
| OTU_420 | blue | K_Bacteria | p_Actinobacteria | c_Actinobacteria | o_Streptosporangiales | f_Streptosporangiales_incertae_sedis | g_Sinosporangium |
| OTU_316 | blue | K_Bacteria | p_Gemmatimonadetes | c_Gemmatimonadetes | o_Gemmatimonadales | f_Gemmatimonadaceae | g_Roseisolibacter |
| OTU_468 | brown | K_Bacteria | p_Acidobacteria | c_Acidobacteriae | o_Gp1 | f_Gp1 | g_Gp1 |
| OTU_499 | turquoise | K_Bacteria | p_Planctomycetes | c_Planctomycetacia | o_Planctomycetales | f_Isosphaeraceae | g_Singulisphaera |
| OTU_827 | yellow | K_Bacteria | p_Proteobacteria | c_Alphaproteobacteria | o_Rhodospirillales | f_Rhodovibrionaceae | g_Tistlia |
| OTU_344 | turquoise | K_Bacteria | p_Firmicutes | c_Bacilli | o_Bacillales | f_Paenibacillaceae 1 | g_Cohnella |
| OTU_336 | turquoise | K_Bacteria | p_Proteobacteria | c_Alphaproteobacteria | o_Micropepsales | f_Micropepsaceae | g_Micropepsis |
| OTU_138 | turquoise | K_Bacteria | p_Chloroflexi | c_Ktedonobacteria | o_Ktedonobacterales | f_Ktedonobacteraceae | g_Ktedonobacter |
| OTU_95 | turquoise | K_Bacteria | p_Proteobacteria | c_Alphaproteobacteria | o_Micropepsales | f_Micropepsaceae | g_Rhizomicrobium |
| OTU_456 | blue | K_Bacteria | p_Proteobacteria | c_Alphaproteobacteria | o_Rhizobiales | f_Hyphomicrobiaceae | g_Hyphomicrobium |
| OTU_199 | brown | K_Bacteria | p_Proteobacteria | c_Alphaproteobacteria | o_Rhodospirillales | f_Reyranellaceae | g_Reyranella |
| OTU_231 | brown | K_Bacteria | p_Acidobacteria | c_Acidobacteriae | o_Acidipila | f_Acidipila | g_Acidipila |
| OTU_76 | green | K_Bacteria | p_Gemmatimonadetes | c_Longimicrobia | o_Longimicrobiales | f_Longimicrobiaceae | g_Longimicrobium |
| OTU_173 | brown | K_Bacteria | p_Proteobacteria | c_Gammaproteobacteria | o_Nevskiales | f_Steroidobacteraceae | g_Povalibacter |
| OTU_258 | turquoise | K_Bacteria | p_Acidobacteria | c_Acidobacteriae | o_Candidatus Koribacter | f_Candidatus Koribacter | g_Candidatus Koribacter |
| OTU_51 | brown | K_Bacteria | p_Proteobacteria | c_Alphaproteobacteria | o_Rhodospirillales | f_Rhodovibrionaceae | g_Tistlia |
| OTU_162 | yellow | K_Bacteria | p_Actinobacteria | c_Actinobacteria | o_Frankiales | f_Frankiaceae | g_Jatrophihabitans |
| OTU_194 | brown | K_Bacteria | p_Proteobacteria | c_Gammaproteobacteria | o_Pseudomonadales | f_Pseudomonadaceae | g_Permianibacter |
| OTU_200 | turquoise | K_Bacteria | p_Proteobacteria | c_Betaproteobacteria | o_Nitrosomonadales | f_Nitrosomonadaceae | g_Nitrosospira |
| OTU_170 | blue | K_Bacteria | p_Chloroflexi | c_Ktedonobacteria | o_Ktedonobacterales | f_Ktedonobacteraceae | g_Ktedonobacter |
| OTU_286 | turquoise | K_Bacteria | p_Firmicutes | c_Bacilli | o_Bacillales | f_Paenibacillaceae 1 | g_Paenibacillus |
| OTU_1697 | blue | K_Bacteria | p_Proteobacteria | c_Alphaproteobacteria | o_Rhizobiales | f_Xanthobacteraceae | g_Xanthobacter |
| OTU_146 | blue | K_Bacteria | p_Proteobacteria | c_Alphaproteobacteria | o_Rhodospirillales | f_Rhodospirillaceae | g_Aliidongia |
| OTU_120 | yellow | K_Bacteria | p_Acidobacteria | c_Acidobacteriae | o_Gp1 | f_Gp1 | g_Gp1 |
| OTU_67 | turquoise | K_Bacteria | p_Gemmatimonadetes | c_Gemmatimonadetes | o_Gemmatimonadales | f_Gemmatimonadaceae | g_Roseisolibacter |
| OTU_2877 | turquoise | K_Bacteria | p_Acidobacteria | c_Acidobacteriae | o_Edaphobacter | f_Edaphobacter | g_Edaphobacter |
| OTU_3427 | turquoise | K_Bacteria | p_Proteobacteria | c_Gammaproteobacteria | o_Gammaproteobacteria_incertae_sedis | f_Acidibacter | g_Acidibacter |
| OTU_218 | blue | K_Bacteria | p_Planctomycetes | c_Planctomycetacia | o_Planctomycetales | f_Gemmataceae | g_Fimbriiglobus |
| OTU_121 | brown | K_Bacteria | p_Proteobacteria | c_Alphaproteobacteria | o_Rhodospirillales | f_Rhodospirillaceae | g_Aliidongia |
| OTU_247 | yellow | K_Bacteria | p_Acidobacteria | c_Acidobacteriae | o_Gp3 | f_Gp3 | g_Gp3 |
| OTU_373 | brown | K_Bacteria | p_Actinobacteria | c_Acidimicrobiia | o_Acidimicrobiales | f_Acidimicrobiales_incertae_sedis | g_Aciditerrimonas |
| OTU_274 | brown | K_Bacteria | p_Actinobacteria | c_Thermoleophilia | o_Gaiellales | f_Gaiellaceae | g_Gaiella |
| OTU_297 | blue | K_Bacteria | p_Actinobacteria | c_Thermoleophilia | o_Gaiellales | f_Gaiellaceae | g_Gaiella |
| OTU_390 | yellow | K_Bacteria | p_Actinobacteria | c_Thermoleophilia | o_Solirubrobacterales | f_Conexibacteraceae | g_Conexibacter |
| OTU_579 | turquoise | K_Bacteria | p_Planctomycetes | c_Planctomycetacia | o_Planctomycetales | f_Gemmataceae | g_Gemmata |
| OTU_77 | turquoise | K_Bacteria | p_Acidobacteria | c_Acidobacteriae | o_Gp1 | f_Gp1 | g_Gp1 |
| OTU_453 | blue | K_Bacteria | p_Proteobacteria | c_Alphaproteobacteria | o_Rhizobiales | f_Bradyrhizobiaceae | g_Pseudolabrys |
| OTU_59 | blue | K_Bacteria | p_Gemmatimonadetes | c_Longimicrobia | o_Longimicrobiales | f_Longimicrobiaceae | g_Longimicrobium |
| OTU_196 | turquoise | K_Bacteria | p_Actinobacteria | c_Actinobacteria | o_Pseudonocardiales | f_Pseudonocardiaceae | g_Umezawaea |
| OTU_86 | blue | K_Bacteria | p_Firmicutes | c_Clostridia | o_Clostridiales | f_Symbiobacteriaceae | g_Caldinitratiruptor |
| OTU_185 | turquoise | K_Bacteria | p_Firmicutes | c_Clostridia | o_Thermosediminibacterales | f_Tepidanaerobacteraceae | g_Biomaibacter |
| OTU_179 | turquoise | K_Bacteria | p_Chloroflexi | c_Ktedonobacteria | o_Ktedonobacterales | f_Ktedonobacteraceae | g_Ktedonobacter |
| OTU_281 | turquoise | K_Bacteria | p_Chloroflexi | c_Ktedonobacteria | o_Ktedonobacterales | f_Ktedonobacteraceae | g_Ktedonobacter |
| OTU_1667 | green | K_Bacteria | p_Acidobacteria | c_Acidobacteriae | o_Gp3 | f_Gp3 | g_Gp3 |
| OTU_404 | blue | K_Bacteria | p_Acidobacteria | c_Acidobacteriae | o_Gp5 | f_Gp5 | g_Gp5 |
| OTU_88 | turquoise | K_Bacteria | p_Proteobacteria | c_Alphaproteobacteria | o_Rhodospirillales | f_Rhodospirillaceae | g_Aliidongia |
| OTU_799 | turquoise | K_Bacteria | p_Acidobacteria | c_Acidobacteriae | o_Gp1 | f_Gp1 | g_Gp1 |
| OTU_239 | green | K_Bacteria | p_Bacteroidetes | c_Cytophagia | o_Cytophagales | f_Cytophagaceae | g_Tellurirhabdus |
| OTU_159 | green | K_Bacteria | p_Acidobacteria | c_Acidobacteriae | o_Gp6 | f_Gp6 | g_Gp6 |
| OTU_139 | brown | K_Bacteria | p_Firmicutes | c_Clostridia | o_Thermosediminibacterales | f_Tepidanaerobacteraceae | g_Biomaibacter |
| OTU_500 | brown | K_Bacteria | p_Acidobacteria | c_Acidobacteriae | o_Gp1 | f_Gp1 | g_Gp1 |
| OTU_945 | green | K_Bacteria | p_Proteobacteria | c_Gammaproteobacteria | o_Acidiferrobacterales | f_Acidiferrobacteraceae | g_Sulfuricaulis |
| OTU_458 | turquoise | K_Bacteria | p_Planctomycetes | c_Planctomycetacia | o_Planctomycetales | f_Isosphaeraceae | g_Singulisphaera |
| OTU_201 | yellow | K_Bacteria | p_Acidobacteria | c_Acidobacteriae | o_Candidatus Solibacter | f_Candidatus Solibacter | g_Candidatus Solibacter |
| OTU_85 | blue | K_Bacteria | p_Proteobacteria | c_Alphaproteobacteria | o_Rhodospirillales | f_Acetobacteraceae | g_Craurococcus |
| OTU_161 | blue | K_Bacteria | p_Proteobacteria | c_Alphaproteobacteria | o_Rhizobiales | f_Kaistiaceae | g_Bauldia |
| OTU_113 | turquoise | K_Bacteria | p_Proteobacteria | c_Alphaproteobacteria | o_Caulobacterales | f_Caulobacteraceae | g_Phenylobacterium |
| OTU_208 | turquoise | K_Bacteria | p_Firmicutes | c_Clostridia | o_Clostridiales | f_Symbiobacteriaceae | g_Caldinitratiruptor |
| OTU_299 | brown | K_Bacteria | p_Chloroflexi | c_Ktedonobacteria | o_Ktedonobacterales | f_Ktedonobacteraceae | g_Ktedonobacter |
| OTU_205 | brown | K_Bacteria | p_Proteobacteria | c_Alphaproteobacteria | o_Rhodospirillales | f_Rhodovibrionaceae | g_Tistlia |
| OTU_3038 | turquoise | K_Bacteria | p_Acidobacteria | c_Acidobacteriae | o_Gp1 | f_Gp1 | g_Gp1 |
| OTU_575 | brown | K_Bacteria | p_Acidobacteria | c_Acidobacteriae | o_Gp3 | f_Gp3 | g_Gp3 |
| OTU_3871 | blue | K_Bacteria | p_Firmicutes | c_Clostridia | o_Thermoanaerobacterales | f_Thermoanaerobacteraceae | g_Syntrophaceticus |
| OTU_147 | turquoise | K_Bacteria | p_Chloroflexi | c_Ktedonobacteria | o_Ktedonobacterales | f_Ktedonobacteraceae | g_Ktedonobacter |
| OTU_457 | brown | K_Bacteria | p_Proteobacteria | c_Betaproteobacteria | o_Burkholderiales | f_Burkholderiaceae | g_Paraburkholderia |
| OTU_463 | yellow | K_Bacteria | p_Acidobacteria | c_Acidobacteriae | o_Candidatus Solibacter | f_Candidatus Solibacter | g_Candidatus Solibacter |
| OTU_181 | yellow | K_Bacteria | p_Planctomycetes | c_Planctomycetacia | o_Planctomycetales | f_Planctomycetaceae | g_Gimesia |
| OTU_131 | turquoise | K_Bacteria | p_Actinobacteria | c_Thermoleophilia | o_Gaiellales | f_Gaiellaceae | g_Gaiella |
| OTU_332 | turquoise | K_Bacteria | p_Firmicutes | c_Bacilli | o_Bacillales | f_Bacillaceae 1 | g_Bacillus |
| OTU_357 | blue | K_Bacteria | p_Proteobacteria | c_Betaproteobacteria | o_Nitrosomonadales | f_Sterolibacteriaceae | g_Georgfuchsia |
| OTU_157 | brown | K_Bacteria | p_Firmicutes | c_Clostridia | o_Clostridiales | f_Symbiobacteriaceae | g_Caldinitratiruptor |
| OTU_224 | turquoise | K_Bacteria | p_Firmicutes | c_Clostridia | o_Clostridiales | f_Symbiobacteriaceae | g_Caldinitratiruptor |
| OTU_416 | blue | K_Bacteria | p_Actinobacteria | c_Thermoleophilia | o_Solirubrobacterales | f_Solirubrobacteraceae | g_Solirubrobacter |
| OTU_78 | brown | K_Bacteria | p_Planctomycetes | c_Planctomycetacia | o_Planctomycetales | f_Gemmataceae | g_Limnoglobus |
| OTU_234 | turquoise | K_Bacteria | p_Acidobacteria | c_Acidobacteriae | o_Gp6 | f_Gp6 | g_Gp6 |
| OTU_298 | blue | K_Bacteria | p_Actinobacteria | c_Acidimicrobiia | o_Acidimicrobiales | f_Acidimicrobiaceae | g_Acidiferrimicrobium |
| OTU_215 | turquoise | K_Bacteria | p_Actinobacteria | c_Actinobacteria | o_Micrococcales | f_Microbacteriaceae | g_Microbacterium |
| OTU_586 | blue | K_Bacteria | p_Actinobacteria | c_Acidimicrobiia | o_Acidimicrobiales | f_Iamiaceae | g_Iamia |
| OTU_324 | blue | K_Bacteria | p_Actinobacteria | c_Actinobacteria | o_Acidothermales | f_Acidothermaceae | g_Acidothermus |
| OTU_634 | blue | K_Bacteria | p_Acidobacteria | c_Acidobacteriae | o_Gp6 | f_Gp6 | g_Gp6 |
| OTU_617 | brown | K_Bacteria | p_Planctomycetes | c_Planctomycetacia | o_Planctomycetales | f_Gemmataceae | g_Fimbriiglobus |
| OTU_260 | yellow | K_Bacteria | p_Actinobacteria | c_Thermoleophilia | o_Solirubrobacterales | f_Conexibacteraceae | g_Conexibacter |
| OTU_187 | turquoise | K_Bacteria | p_Firmicutes | c_Clostridia | o_Clostridiales | f_Ruminococcaceae | g_Monoglobus |
| OTU_521 | blue | K_Bacteria | p_Actinobacteria | c_Actinobacteria | o_Micromonosporales | f_Micromonosporaceae | g_Rugosimonospora |
| OTU_154 | turquoise | K_Bacteria | p_Proteobacteria | c_Alphaproteobacteria | o_Rhodospirillales | f_Rhodospirillaceae | g_Aliidongia |
| OTU_188 | turquoise | K_Bacteria | p_Chloroflexi | c_Ktedonobacteria | o_Ktedonobacterales | f_Ktedonobacteraceae | g_Ktedonobacter |
| OTU_149 | turquoise | K_Bacteria | p_Actinobacteria | c_Actinobacteria | o_Pseudonocardiales | f_Pseudonocardiaceae | g_Pseudonocardia |
| OTU_765 | yellow | K_Bacteria | p_Proteobacteria | c_Deltaproteobacteria | o_Syntrophobacterales | f_Syntrophobacteraceae | g_Desulfoglaeba |
| OTU_492 | blue | K_Bacteria | p_Actinobacteria | c_Acidimicrobiia | o_Acidimicrobiales | f_Acidimicrobiales_incertae_sedis | g_Aciditerrimonas |
| OTU_768 | turquoise | K_Bacteria | p_Firmicutes | c_Bacilli | o_Bacillales | f_Bacillaceae 1 | g_Bacillus |
| OTU_384 | yellow | K_Bacteria | p_Bacteroidetes | c_Cytophagia | o_Cytophagales | f_Fulvivirgaceae | g_Chryseolinea |
| OTU_800 | turquoise | K_Bacteria | p_Actinobacteria | c_Actinobacteria | o_Streptomycetales | f_Streptomycetaceae | g_Streptomyces |
| OTU_477 | brown | K_Bacteria | p_Acidobacteria | c_Acidobacteriae | o_Gp3 | f_Gp3 | g_Gp3 |
| OTU_1276 | turquoise | K_Bacteria | p_Firmicutes | c_Bacilli | o_Bacillales | f_Paenibacillaceae 1 | g_Cohnella |
| OTU_928 | blue | K_Bacteria | p_Proteobacteria | c_Alphaproteobacteria | o_Rhodospirillales | f_Reyranellaceae | g_Reyranella |
| OTU_540 | yellow | K_Bacteria | p_Gemmatimonadetes | c_Gemmatimonadetes | o_Gemmatimonadales | f_Gemmatimonadaceae | g_Gemmatirosa |
| OTU_180 | brown | K_Bacteria | p_Chloroflexi | c_Ktedonobacteria | o_Ktedonobacterales | f_Ktedonobacteraceae | g_Ktedonobacter |
| OTU_354 | turquoise | K_Bacteria | p_Actinobacteria | c_Thermoleophilia | o_Solirubrobacterales | f_Conexibacteraceae | g_Conexibacter |
| OTU_317 | blue | K_Bacteria | p_Proteobacteria | c_Betaproteobacteria | o_Nitrosomonadales | f_Sterolibacteriaceae | g_Denitratisoma |
| OTU_414 | turquoise | K_Bacteria | p_Proteobacteria | c_Alphaproteobacteria | o_Rhodospirillales | f_Acetobacteraceae | g_Rhodopila |
| OTU_263 | blue | K_Bacteria | p_Proteobacteria | c_Deltaproteobacteria | o_Myxococcales | f_Cystobacteraceae | g_Cystobacter |
| OTU_678 | turquoise | K_Bacteria | p_Verrucomicrobia | c_Subdivision3 | o_Limisphaera | f_Limisphaera | g_Limisphaera |
| OTU_401 | turquoise | K_Bacteria | p_Proteobacteria | c_Alphaproteobacteria | o_Micropepsales | f_Micropepsaceae | g_Rhizomicrobium |
| OTU_1150 | blue | K_Bacteria | p_Proteobacteria | c_Deltaproteobacteria | o_Myxococcales | f_Haliangiaceae | g_Haliangium |
| OTU_3246 | turquoise | K_Bacteria | p_Proteobacteria | c_Deltaproteobacteria | o_Desulfobacterales | f_Desulfobacteraceae | g_Algorimarina |
| OTU_256 | turquoise | K_Bacteria | p_Chloroflexi | c_Ktedonobacteria | o_Thermogemmatisporales | f_Thermogemmatisporaceae | g_Thermogemmatispora |
| OTU_2175 | blue | K_Bacteria | p_Actinobacteria | c_Thermoleophilia | o_Solirubrobacterales | f_Baekduiaceae | g_Baekduia |
| OTU_377 | blue | K_Bacteria | p_Proteobacteria | c_Alphaproteobacteria | o_Rhizobiales | f_Devosiaceae | g_Devosia |
| OTU_2544 | blue | K_Bacteria | p_Actinobacteria | c_Actinobacteria | o_Micrococcales | f_Intrasporangiaceae | g_Terrabacter |
| OTU_567 | blue | K_Bacteria | p_Bacteroidetes | c_Chitinophagia | o_Chitinophagales | f_Chitinophagaceae | g_Puia |
| OTU_513 | yellow | K_Bacteria | p_Proteobacteria | c_Alphaproteobacteria | o_Micropepsales | f_Micropepsaceae | g_Rhizomicrobium |
| OTU_283 | turquoise | K_Bacteria | p_Chloroflexi | c_Ktedonobacteria | o_Ktedonobacterales | f_Ktedonobacteraceae | g_Ktedonobacter |
| OTU_3523 | blue | K_Bacteria | p_Proteobacteria | c_Alphaproteobacteria | o_Rhizobiales | f_Bradyrhizobiaceae | g_Pseudorhodoplanes |
| OTU_280 | brown | K_Bacteria | p_Firmicutes | c_Bacilli | o_Bacillales | f_Bacillaceae 1 | g_Bacillus |
| OTU_367 | yellow | K_Bacteria | p_Proteobacteria | c_Deltaproteobacteria | o_Myxococcales | f_Haliangiaceae | g_Haliangium |
| OTU_310 | blue | K_Bacteria | p_Acidobacteria | c_Acidobacteriae | o_Gp1 | f_Gp1 | g_Gp1 |
| OTU_2691 | blue | K_Bacteria | p_Proteobacteria | c_Alphaproteobacteria | o_Rhizobiales | f_Bradyrhizobiaceae | g_Afipia |
| OTU_348 | turquoise | K_Bacteria | p_Actinobacteria | c_Actinobacteria | o_Mycobacteriales | f_Nocardiaceae | g_Nocardia |
| OTU_396 | turquoise | K_Bacteria | p_Actinobacteria | c_Actinobacteria | o_Catenulisporales | f_Catenulisporaceae | g_Catenulispora |
| OTU_262 | green | K_Bacteria | p_Acidobacteria | c_Acidobacteriae | o_Gp3 | f_Gp3 | g_Gp3 |
| OTU_333 | turquoise | K_Bacteria | p_Proteobacteria | c_Alphaproteobacteria | o_Rhodospirillales | f_Acetobacteraceae | g_Humitalea |
| OTU_323 | brown | K_Bacteria | p_Actinobacteria | c_Acidimicrobiia | o_Acidimicrobiales | f_Acidimicrobiales_incertae_sedis | g_Aciditerrimonas |
| OTU_3382 | turquoise | K_Bacteria | p_Actinobacteria | c_Acidimicrobiia | o_Acidimicrobiales | f_Acidimicrobiaceae | g_Acidiferrimicrobium |
| OTU_240 | turquoise | K_Bacteria | p_Chloroflexi | c_Ktedonobacteria | o_Ktedonobacterales | f_Ktedonobacteraceae | g_Ktedonobacter |
| OTU_206 | brown | K_Bacteria | p_Proteobacteria | c_Betaproteobacteria | o_Burkholderiales | f_Oxalobacteraceae | g_Collimonas |
| OTU_111 | turquoise | K_Bacteria | p_Proteobacteria | c_Alphaproteobacteria | o_Rhodospirillales | f_Rhodospirillaceae | g_Aliidongia |
| OTU_464 | turquoise | K_Bacteria | p_Acidobacteria | c_Acidobacteriae | o_Gp3 | f_Gp3 | g_Gp3 |
| OTU_433 | blue | K_Bacteria | p_Actinobacteria | c_Acidimicrobiia | o_Acidimicrobiales | f_Acidimicrobiales_incertae_sedis | g_Aciditerrimonas |
| OTU_190 | yellow | K_Bacteria | p_Proteobacteria | c_Alphaproteobacteria | o_Rhodospirillales | f_Rhodospirillaceae | g_Aliidongia |
| OTU_576 | blue | K_Bacteria | p_Acidobacteria | c_Acidobacteriae | o_Gp5 | f_Gp5 | g_Gp5 |
| OTU_308 | yellow | K_Bacteria | p_Proteobacteria | c_Betaproteobacteria | o_Nitrosomonadales | f_Sterolibacteriaceae | g_Sterolibacterium |
| OTU_315 | yellow | K_Bacteria | p_Proteobacteria | c_Alphaproteobacteria | o_Rhodospirillales | f_Rhodospirillaceae | g_Lacibacterium |
| OTU_434 | green | K_Bacteria | p_Chloroflexi | c_Ktedonobacteria | o_Ktedonobacterales | f_Ktedonobacteraceae | g_Ktedonobacter |
| OTU_436 | blue | K_Bacteria | p_Proteobacteria | c_Deltaproteobacteria | o_Syntrophobacterales | f_Syntrophobacteraceae | g_Desulfoglaeba |
| OTU_727 | yellow | K_Bacteria | p_Proteobacteria | c_Alphaproteobacteria | o_Rhizobiales | f_Beijerinckiaceae | g_Beijerinckia |
| OTU_252 | brown | K_Bacteria | p_Acidobacteria | c_Acidobacteriae | o_Gp7 | f_Gp7 | g_Gp7 |
| OTU_284 | turquoise | K_Bacteria | p_Acidobacteria | c_Acidobacteriae | o_Gp1 | f_Gp1 | g_Gp1 |
| OTU_294 | turquoise | K_Bacteria | p_Actinobacteria | c_Actinobacteria | o_Catenulisporales | f_Actinospicaceae | g_Actinocrinis |
| OTU_441 | brown | K_Bacteria | p_Acidobacteria | c_Acidobacteriae | o_Gp1 | f_Gp1 | g_Gp1 |
| OTU_397 | turquoise | K_Bacteria | p_Chloroflexi | c_Ktedonobacteria | o_Ktedonobacterales | f_Ktedonobacteraceae | g_Ktedonobacter |
| OTU_447 | brown | K_Bacteria | p_Firmicutes | c_Bacilli | o_Bacillales | f_Alicyclobacillaceae | g_Tumebacillus |
| OTU_4003 | turquoise | K_Bacteria | p_Chloroflexi | c_Ktedonobacteria | o_Ktedonobacterales | f_Ktedonobacteraceae | g_Ktedonobacter |
| OTU_176 | brown | K_Bacteria | p_Proteobacteria | c_Alphaproteobacteria | o_Rhodospirillales | f_Acetobacteraceae | g_Acidisoma |
| OTU_620 | turquoise | K_Bacteria | p_Chloroflexi | c_Ktedonobacteria | o_Ktedonobacterales | f_Ktedonobacteraceae | g_Ktedonobacter |
| OTU_418 | turquoise | K_Bacteria | p_Planctomycetes | c_Planctomycetacia | o_Planctomycetales | f_Planctomycetaceae | g_Schlesneria |
| OTU_171 | turquoise | K_Bacteria | p_Chloroflexi | c_Ktedonobacteria | o_Ktedonobacterales | f_Ktedonobacteraceae | g_Ktedonobacter |
| OTU_251 | green | K_Bacteria | p_Actinobacteria | c_Thermoleophilia | o_Solirubrobacterales | f_Paraconexibacteraceae | g_Paraconexibacter |
| OTU_424 | turquoise | K_Bacteria | p_Chloroflexi | c_Caldilineae | o_Caldilineales | f_Caldilineaceae | g_Litorilinea |
| OTU_715 | blue | K_Bacteria | p_Gemmatimonadetes | c_Gemmatimonadetes | o_Gemmatimonadales | f_Gemmatimonadaceae | g_Gemmatirosa |
| OTU_355 | yellow | K_Bacteria | p_Planctomycetes | c_Planctomycetacia | o_Planctomycetales | f_Gemmataceae | g_Gemmata |
| OTU_470 | turquoise | K_Bacteria | p_Chloroflexi | c_Ktedonobacteria | o_Ktedonobacterales | f_Ktedonobacteraceae | g_Ktedonobacter |
| OTU_109 | blue | K_Bacteria | p_Proteobacteria | c_Alphaproteobacteria | o_Rhodospirillales | f_Rhodospirillaceae | g_Aliidongia |
| OTU_3218 | yellow | K_Bacteria | p_Acidobacteria | c_Acidobacteriae | o_Gp2 | f_Gp2 | g_Gp2 |
| OTU_462 | blue | K_Bacteria | p_Actinobacteria | c_Actinobacteria | o_Propionibacteriales | f_Nocardioidaceae | g_Nocardioides |
| OTU_490 | blue | K_Bacteria | p_Actinobacteria | c_Thermoleophilia | o_Gaiellales | f_Gaiellaceae | g_Gaiella |
| OTU_3907 | blue | K_Bacteria | p_Planctomycetes | c_Planctomycetacia | o_Planctomycetales | f_Planctomycetaceae | g_Gimesia |
| OTU_4304 | blue | K_Bacteria | p_Acidobacteria | c_Acidobacteriae | o_Terracidiphilus | f_Terracidiphilus | g_Terracidiphilus |
| OTU_186 | brown | K_Bacteria | p_Planctomycetes | c_Planctomycetacia | o_Planctomycetales | f_Gemmataceae | g_Fimbriiglobus |
| OTU_243 | turquoise | K_Bacteria | p_Proteobacteria | c_Alphaproteobacteria | o_Rhizobiales | f_Pleomorphomonadaceae | g_Hartmannibacter |
| OTU_739 | blue | K_Bacteria | p_Actinobacteria | c_Actinobacteria | o_Nakamurellales | f_Nakamurellaceae | g_Nakamurella |
| OTU_207 | brown | K_Bacteria | p_Planctomycetes | c_Planctomycetacia | o_Planctomycetales | f_Isosphaeraceae | g_Singulisphaera |
| OTU_430 | yellow | K_Bacteria | p_Acidobacteria | c_Acidobacteriae | o_Gp13 | f_Gp13 | g_Gp13 |
| OTU_494 | turquoise | K_Bacteria | p_Firmicutes | c_Bacilli | o_Bacillales | f_Paenibacillaceae 1 | g_Paenibacillus |
| OTU_580 | turquoise | K_Bacteria | p_Actinobacteria | c_Actinobacteria | o_Streptosporangiales | f_Thermomonosporaceae | g_Actinoallomurus |
| OTU_391 | blue | K_Bacteria | p_Chloroflexi | c_Chloroflexia | o_Kallotenuales | f_Kallotenuaceae | g_Kallotenue |
| OTU_1036 | brown | K_Bacteria | p_Proteobacteria | c_Alphaproteobacteria | o_Caulobacterales | f_Caulobacteraceae | g_Phenylobacterium |
| OTU_362 | blue | K_Bacteria | p_Actinobacteria | c_Acidimicrobiia | o_Acidimicrobiales | f_Acidimicrobiales_incertae_sedis | g_Aciditerrimonas |
| OTU_352 | yellow | K_Bacteria | p_Proteobacteria | c_Alphaproteobacteria | o_Rhodospirillales | f_Acetobacteraceae | g_Humitalea |
| OTU_174 | yellow | K_Bacteria | p_Chloroflexi | c_Ktedonobacteria | o_Ktedonobacterales | f_Ktedonobacteraceae | g_Ktedonobacter |
| OTU_716 | blue | K_Bacteria | p_Proteobacteria | c_Betaproteobacteria | o_Nitrosomonadales | f_Nitrosomonadaceae | g_Nitrosospira |
| OTU_213 | blue | K_Bacteria | p_Proteobacteria | c_Alphaproteobacteria | o_Rhizobiales | f_Phyllobacteriaceae | g_Aminobacter |
| OTU_287 | blue | K_Bacteria | p_Proteobacteria | c_Alphaproteobacteria | o_Rhodospirillales | f_Rhodospirillaceae | g_Aliidongia |
| OTU_363 | green | K_Bacteria | p_Proteobacteria | c_Alphaproteobacteria | o_Rhodospirillales | f_Acetobacteraceae | g_Acidiphilium |
| OTU_295 | turquoise | K_Bacteria | p_Acidobacteria | c_Acidobacteriae | o_Gp2 | f_Gp2 | g_Gp2 |
| OTU_238 | turquoise | K_Bacteria | p_Chloroflexi | c_Ktedonobacteria | o_Ktedonobacterales | f_Ktedonobacteraceae | g_Ktedonobacter |
| OTU_130 | green | K_Bacteria | p_Chloroflexi | c_Ktedonobacteria | o_Ktedonobacterales | f_Ktedonobacteraceae | g_Ktedonobacter |
| OTU_361 | brown | K_Bacteria | p_Verrucomicrobia | c_Opitutae | o_Opitutales | f_Opitutaceae | g_Lacunisphaera |
| OTU_1769 | turquoise | K_Bacteria | p_Actinobacteria | c_Actinobacteria | o_Streptosporangiales | f_Thermomonosporaceae | g_Actinoallomurus |
| OTU_859 | blue | K_Bacteria | p_Planctomycetes | c_Planctomycetacia | o_Planctomycetales | f_Planctomycetaceae | g_Gimesia |
| OTU_2991 | grey | K_Bacteria | p_Acidobacteria | c_Acidobacteriae | o_Gp6 | f_Gp6 | g_Gp6 |
| OTU_838 | turquoise | K_Bacteria | p_Bacteroidetes | c_Chitinophagia | o_Chitinophagales | f_Chitinophagaceae | g_Flavitalea |
| OTU_264 | turquoise | K_Bacteria | p_Proteobacteria | c_Alphaproteobacteria | o_Rhodospirillales | f_Acetobacteraceae | g_Rhodovastum |
| OTU_1717 | turquoise | K_Bacteria | p_Planctomycetes | c_Planctomycetacia | o_Planctomycetales | f_Gemmataceae | g_Fimbriiglobus |
| FUN_4 | turquoise | K_Fungi | p_Ascomycota | c_Leotiomycetes | o_Helotiales | f_Hyaloscyphaceae | g_Hyaloscyphaceae_unidentified |
| FUN_2 | turquoise | K_Fungi | p_Zygomycota | c_Incertae_sedis_10 | o_Mortierellales | f_Mortierellaceae | g_Mortierella |
| FUN_11 | green | K_Fungi | p_Ascomycota | c_Eurotiomycetes | o_Eurotiales | f_Trichocomaceae | g_Trichocomaceae_unidentified |
| FUN_9 | green | K_Fungi | p_Basidiomycota | c_Wallemiomycetes | o_Geminibasidiales | f_Geminibasidiaceae | g_Geminibasidium |
| FUN_13 | grey | K_Fungi | p_Ascomycota | c_Eurotiomycetes | o_Chaetothyriales | f_Herpotrichiellaceae | g_Cladophialophora |
| FUN_64 | turquoise | K_Fungi | p_Ascomycota | c_Eurotiomycetes | o_Chaetothyriales | f_Chaetothyriales_unidentified | g_Chaetothyriales_unidentified_1 |
| FUN_25 | turquoise | K_Fungi | p_Ascomycota | c_Eurotiomycetes | o_Eurotiales | f_Trichocomaceae | g_Talaromyces |
| FUN_146 | grey | K_Fungi | p_Ascomycota | c_Sordariomycetes | o_Sordariales | f_Chaetomiaceae | g_Chaetomium |
| FUN_1 | turquoise | K_Fungi | p_Ascomycota | c_Sordariomycetes | o_Ophiostomatales | f_Ophiostomataceae | g_Ophiostomataceae_unidentified |
| FUN_10 | green | K_Fungi | p_Zygomycota | c_Incertae_sedis_10 | o_Mortierellales | f_Mortierellaceae | g_Mortierella |
| FUN_913 | blue | K_Fungi | p_Basidiomycota | c_Wallemiomycetes | o_Geminibasidiales | f_Geminibasidiaceae | g_Geminibasidium |
| FUN_40 | yellow | K_Fungi | p_Ascomycota | c_Sordariomycetes | o_Coniochaetales | f_Coniochaetaceae | g_Lecythophora |
| FUN_8 | blue | K_Fungi | p_Ascomycota | c_Sordariomycetes | o_Hypocreales | f_Incertae_sedis_3 | g_Ilyonectria |
| FUN_94 | turquoise | K_Fungi | p_Ascomycota | c_Eurotiomycetes | o_Eurotiales | f_Trichocomaceae | g_Penicillium |
| FUN_28 | grey | K_Fungi | p_Ascomycota | c_Dothideomycetes | o_Incertae_sedis_8 | f_Myxotrichaceae | g_Oidiodendron |
| FUN_1610 | yellow | K_Fungi | p_Ascomycota | c_Eurotiomycetes | o_Eurotiales | f_Trichocomaceae | g_Talaromyces |
| FUN_59 | yellow | K_Fungi | p_Ascomycota | c_Leotiomycetes | o_Helotiales | f_Helotiales_unidentified | g_Helotiales_unidentified_1 |
| FUN_66 | brown | K_Fungi | p_Ascomycota | c_Sordariomycetes | o_Microascales | f_Microascaceae | g_Scedosporium |
| FUN_26 | yellow | K_Fungi | p_Ascomycota | c_Sordariomycetes | o_Hypocreales | f_Nectriaceae | g_Fusarium |
| FUN_39 | blue | K_Fungi | p_Ascomycota | c_Eurotiomycetes | o_Eurotiales | f_Trichocomaceae | g_Penicillium |
| FUN_23 | green | K_Fungi | p_Ascomycota | c_Leotiomycetes | o_Helotiales | f_Helotiales_unidentified | g_Helotiales_unidentified_1 |
| FUN_70 | grey | K_Fungi | p_Ascomycota | c_Eurotiomycetes | o_Eurotiales | f_Trichocomaceae | g_Talaromyces |
| FUN_12 | brown | K_Fungi | p_Ascomycota | c_Leotiomycetes | o_Helotiales | f_Helotiales_unidentified | g_Helotiales_unidentified_1 |
| FUN_85 | grey | K_Fungi | p_Ascomycota | c_Eurotiomycetes | o_Eurotiales | f_Trichocomaceae | g_Aspergillus |
| FUN_27 | brown | K_Fungi | p_Ascomycota | c_Sordariomycetes | o_Sordariales | f_Chaetomiaceae | g_Chaetomiaceae_unidentified |
| FUN_63 | blue | K_Fungi | p_Ascomycota | c_Sordariomycetes | o_Hypocreales | f_Ophiocordycipitaceae | g_Purpureocillium |
| FUN_56 | yellow | K_Fungi | p_Ascomycota | c_Sordariomycetes | o_Hypocreales | f_Hypocreaceae | g_Trichoderma |
| FUN_71 | yellow | K_Fungi | p_Ascomycota | c_Sordariomycetes | o_Hypocreales | f_Hypocreaceae | g_Trichoderma |
| FUN_55 | green | K_Fungi | p_Ascomycota | c_Eurotiomycetes | o_Chaetothyriales | f_Herpotrichiellaceae | g_Cladophialophora |
| FUN_30 | yellow | K_Fungi | p_Basidiomycota | c_Agaricomycetes | o_Agaricales | f_Agaricaceae | g_Agaricaceae_unidentified |
| FUN_69 | yellow | K_Fungi | p_Ascomycota | c_Ascomycota_unidentified | o_Ascomycota_unidentified_1 | f_Ascomycota_unidentified_1 | g_Ascomycota_unidentified_1_1 |
| FUN_58 | brown | K_Fungi | p_Ascomycota | c_Eurotiomycetes | o_Eurotiales | f_Trichocomaceae | g_Trichocomaceae_unidentified |
| FUN_163 | blue | K_Fungi | p_Ascomycota | c_Eurotiomycetes | o_Eurotiales | f_Trichocomaceae | g_Aspergillus |
| FUN_139 | turquoise | K_Fungi | p_Ascomycota | c_Eurotiomycetes | o_Eurotiales | f_Trichocomaceae | g_Trichocomaceae_unidentified |
| FUN_36 | grey | K_Fungi | p_Zygomycota | c_Incertae_sedis_10 | o_Mucorales | f_Umbelopsidaceae | g_Umbelopsis |
| FUN_211 | green | K_Fungi | p_Ascomycota | c_Dothideomycetes | o_Pleosporales | f_Pleosporales_unidentified | g_Pleosporales_unidentified_1 |
| FUN_151 | blue | K_Fungi | p_Ascomycota | c_Ascomycota_unidentified | o_Ascomycota_unidentified_1 | f_Ascomycota_unidentified_1 | g_Ascomycota_unidentified_1_1 |
| FUN_18 | brown | K_Fungi | p_Ascomycota | c_Leotiomycetes | o_Helotiales | f_Helotiales_unidentified | g_Helotiales_unidentified_1 |
| FUN_91 | green | K_Fungi | p_Basidiomycota | c_Agaricomycetes | o_Agaricales | f_Strophariaceae | g_Gymnopilus |
| FUN_46 | turquoise | K_Fungi | p_Ascomycota | c_Dothideomycetes | o_Pleosporales | f_Sporormiaceae | g_Westerdykella |
| FUN_16 | brown | K_Fungi | p_Ascomycota | c_Sordariomycetes | o_Microascales | f_Microascaceae | g_Pseudallescheria |
| FUN_5 | yellow | K_Fungi | p_Ascomycota | c_Sordariomycetes | o_Hypocreales | f_Nectriaceae | g_Flagellospora |
| FUN_100 | blue | K_Fungi | p_Ascomycota | c_Dothideomycetes | o_Pleosporales | f_Pleosporales_unidentified | g_Pleosporales_unidentified_1 |

Table S2 Soil ecosystem multifunctionality indexes across the treatments in the *C. camphora* coppice planting.

| Variables | RZ | TZ | AL | Categories |
| --- | --- | --- | --- | --- |
| SOC (g·kg^-1^) | 19.56±1.55a | 12.60±1.62b | 13.55±0.5ab | soil nutrient cycling |
| TN (g·kg^-1^) | 1.78±0.21a | 1.14±0.11b | 0.99±0.04b | soil nutrient cycling |
| AN (g·kg^-1^) | 212.62±24.96a | 158.75±18.79b | 118.48±9.6c | soil fertility |
| AP (g·kg^-1^) | 32.54±6.42a | 6.86±0.65b | 4.93±0.54b | soil fertility |
| INV (mg·g^-1^·24h^-1^) | 6.56±1.97a | 6.10±1.30a | 4.43±0.66a | soil activity |
| UE（μg·g^-1^·24h^-1^） | 342±35a | 247±18b | 212±10b | soil activity |
| ACP（μmol·g^-1^·24h^-1^） | 20.48±1.85b | 18.97±1.49b | 23.53±0.81a | soil activity |
| CAT（mg·g^-1^·24h^-1^） | 1.14±0.07a | 0.70±0.08b | 1.05±0.03a | soil activity |
| PPO（mg·g^-1^·24h^-1^） | 30.06±4.24a | 23.88±0.64b | 14.74±3.9c | soil activity |
| POD（mg·g^-1^·24h^-1^） | 25.36±5.35a | 16.58±4.45b | 2.68±0.01c | soil activity |

AL: abandoned land, RZ: root zone, TZ: transition zone. SOC: soil organic carbon, TN: total nitrogen, AN: alkaline nitrogen, AP: available phosphorus; INV: invertase, UE: urease, ACP: acid phosphatase, CAT: catalase, PPO: polyphenol oxidase, POD: peroxidase

Table S3 Difference in soil core microbiota based on the similarity test of ANOSIM.

| Treatment | Soil core microbiota | |
| --- | --- | --- |
|  | *r* | *p* |
| AL vs RZ | **0.9272** | **0.027** |
| AL vs TZ | **0.9271** | **0.030** |
| RZ vs TZ | **0.8438** | **0.026** |

AL: abandoned land, RZ: root zone, TZ: transition zone. Significant values are highlighted in bold.
